# Spin-related symmetry breaking induced by half-disordered hybridization in Bi_x_Er_2-x_Ru_2_O_7_ pyrochlores for acidic oxygen evolution

**DOI:** 10.1038/s41467-022-31874-4

**Published:** 2022-07-15

**Authors:** Gang Zhou, Peifang Wang, Bin Hu, Xinyue Shen, Chongchong Liu, Weixiang Tao, Peilin Huang, Lizhe Liu

**Affiliations:** 1 Key Laboratory of Integrated Regulation and Resource Development on Shallow Lake of Ministry of Education, College of Environment, Hohai University, Nanjing, 210098 People’s Republic of China; 2 College of Electronic and Optical Engineering, Nanjing University of Posts and Telecommunications, Nanjing, 210023 People’s Republic of China; 3 Jiangsu Key Laboratory for Nanotechnology and Collaborative Innovation Center of Advanced Microstructures, National Laboratory of Solid State Microstructures, Nanjing University, Nanjing, 210093 People’s Republic of China; 4 Guangxi Key Laboratory of Nuclear Physics and Nuclear Technology, Guangxi Normal University, Guilin, 514004 People’s Republic of China

**Keywords:** Electrocatalysis, Nanoscale materials, Catalyst synthesis

## Abstract

While acidic oxygen evolution reaction plays a critical role in electrochemical energy conversion devices, the sluggish reaction kinetics and poor stability in acidic electrolyte challenges materials development. Unlike traditional nano-structuring approaches, this work focuses on the structural symmetry breaking to rearrange spin electron occupation and optimize spin-dependent orbital interaction to alter charge transfer between catalysts and reactants. Herein, we propose an atomic half-disordering strategy in multistage-hybridized Bi_x_Er_2-x_Ru_2_O_7_ pyrochlores to reconfigure orbital degeneracy and spin-related electron occupation. This strategy involves controlling the bonding interaction of Bi-6*s* lone pair electrons, in which partial atom rearrangement makes the active sites transform into asymmetric high-spin states from symmetric low-spin states. As a result, the half-disordered Bi_x_Er_2-x_Ru_2_O_7_ pyrochlores demonstrate an overpotential of ~0.18 V at 10 mA cm^−2^ accompanied with excellent stability of 100 h in acidic electrolyte. Our findings not only provide a strategy for designing atom-disorder-related catalysts, but also provides a deeper understanding of the spin-related acidic oxygen evolution reaction kinetics.

## Abstract (web summary)

While water electrolysis offers a potential path for renewable hydrogen fuel, water oxidation electrocatalysts typically suffer from poor stabilities in acid. Here, authors prepare ruthenium-based pyrochlores and demonstrate promising activities and durabilities for acidic water electro-oxidation.

## Introduction

The massive economic development and fast depletion of fossil energies demands sustainable strategies to exploit carbon neutral fuels using clean electricity. Based on this consideration, water electrolysis has been considered as a feasible approach to obtain clean and renewable hydrogen, but is facing a grand challenge of the sluggish reaction kinetics for oxygen evolution reaction (OER) and poor electrochemical chemical stability in acidic electrolyte^1,2^. Compared to the alkaline conditions, the OER in acidic electrolyte is much more desirable because water acidic electrolysis has faster reaction rate, higher ionic conductivity, and wider partial load range^3,4^. Therefore, various catalysts such as perovskites, transition metal oxides, and layered nanostructures have been proposed to improve OER performance^3–6^. Unfortunately, most of the known active metal oxides demonstrate inferior reaction activities in acidic media than alkaline condition and they also cannot keep an excellent electrochemical stability during harsh acidic operation^3,7^. Compared with the most of acidic catalysts, Ru-based catalysts usually possess a higher catalytic activity in acidic electrolyte because of suitable binding interaction between intermediates and surface active site^8^, but with a lower electrochemical stability. Therefore, recent designs about acidic catalysts must consider the tradeoff between reaction activity and structural stability.

Fundamentally, the lack of stability of Ru-based materials in acidic electrolyte is mainly attributed to the over-oxidation of exposed Ru sites during OER, leading to a valence transition from Ru^4+^ to Ru^>4+^ at high overpotential^9,10^. The generation of soluble high valence Ru^>4+^ derivatives unavoidably cause the collapse of the crystal structure, deteriorating the electrochemical stability. Consequently, we can speculate that the detrimental over-oxidation of Ru-based electrocatalysts can be effectively hindered in OER application if we can regulate the electronic structure of catalysts to make the bonding strength between Ru site and the coordinated anions stronger than the redox H_2_O/O_2_ energy. However, looking from catalytic kinetics, the overprotection on Ru site can enhance the structural stability but not benefits for the adsorption and dissociation of intermediates, leading to a lower reactive activity. Generally speaking, current acidic OER mainly focus on the thermodynamic process to realize a tradeoff between reaction activity and structural stability^11–19^, but the spin-dependent orbital interaction between active sites and intermediates has been unintentionally neglected. In view of this, the spin catalysis concept can be proposed to simultaneously improve the structural stability and catalytic activity by regulating spin electron configuration^20^, in which the spin-down states can be used to hinder over-oxidation meanwhile the spin-up states can support the higher reactive activity. Therefore, developing a feasible strategy to regulate spin electron configuration needs to be considered.

In principle, the material’s intrinsic physical properties are mainly determined by the crystal symmetry and atomic distribution. If the room-temperature thermal disturbance can cause an atomic disordered-hybridization to reduce the crystal symmetry, some nondegenerate orbitals can be opened to trigger a spin electron reconfiguration. Inspired by this consideration, spin-related symmetry breaking induced by atomic disordered-hybridization becomes an intriguing and feasible strategy to design new-type catalysts with higher reactive activity and electrochemical stability. It is important to note that this proposal is completely different from traditional doping strategy as recent reports^3,11,16,19^. They are as follows: (1) the host elements can be partially replaced by traditional doping atoms but that cannot lead to a controllable structural symmetry breaking; (2) the metal cations acting as active sites have been completely coordinated by the nearest-neighboring anions, which makes it cannot directly generate super-exchange interaction with metal dopants. Therefore, the influence of traditional doping strategy onto orbital degeneracy and spin electron occupation is too limited, which needs the catalysts to simultaneously possess two factors that are opposite response to symmetry breaking.

In this work, to realize our suggestion, the Bi_x_Er_2-x_Ru_2_O_7_ pyrochlores (BERO) are fabricated to disclose the correlation between spin-related symmetry breaking and OER performance in acidic electrolyte. This is because that the Bi-6*s* lone pair electrons can affect the bonding interaction of Ru site with the nearest-neighbor coordinated oxygen atoms and lead to an atomic disordered-hybridization at room temperature^21^, which can make RuO_6_ coordination polyhedron in Bi_2_Ru_2_O_7_ (BRO) change into D_3d_ symmetry from O_h_ point group by electron-phonon interaction. Distinguished from other conventional pyrochlores^11,13–19,22^ with obvious atomic disorder feature, the Er_2_Ru_2_O_7_ system becomes abnormal and complicated due to their particular quantum exchange interaction between Er and Ru ions at d and f orbitals. The inverse uniform magnetic susceptibility measured as a function of temperature displays a linear change and Ru and Er sublattice keep an ordering configuration at room temperature^23^, which is obviously different from the other ruthenium pyrochlores with disordering structures^24^. More importantly, in Er_2_Ru_2_O_7_ sample, the magnetic and structural unit cells are of the same size and 16-fold degenerate ground state of the Er ion splits into 8 Kramers doublets. The neutron scattering data demonstrate that some additionally intensity can be seen on top of the existing Bragg peaks (Q = 0)^23^, which is associated with ordered structure Ru sublattice. This ordering of Er and Ru sublattice have never been observed in others pyrochlores because the face centered cubic symmetry in Er_2_Ru_2_O_7_ is conserved. Therefore, the introduced Er cations in BRO are not sensitive to this symmetry breaking, which can be used to control the atomic disorder degree in Bi_x_Er_2-x_Ru_2_O_7_ sample (named as half-disorder). As atomic disordering operation to eliminate orbital degeneracy, the spin electron configuration can be regulated with symmetry-breaking-generated orbital splitting. The relative electrochemical tests demonstrate that this multistage-hybridized Bi_x_Er_2-x_Ru_2_O_7_ can demonstrate a super-low overpotential of ~0.18 V at 10 mA cm^−2^ accompanied with excellent stability of 100 h in acidic electrolyte. This work provides a new insight into understanding the contribution of spin-related symmetry breaking induced by atomic half-disordered hybridization onto improving catalytic activity and electrochemical stability for OER in acidic electrolyte.

## Results

### Reaction mechanism design and physical characterization

**Fig. 1:**
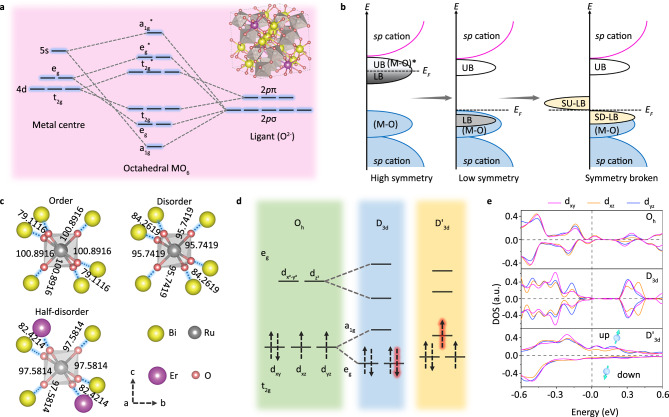
Reaction mechanism design. **a** The molecular orbital energy diagram for octahedral MO_6_. **b** Schematic formation of energy splitting and electronic structure transformation, E_F_ represents the Fermi level. **c** The RuO_6_ coordination polyhedron in different BERO samples with ordered, disordered and half-disordered configuration. **d** Ru-4*d* orbital splitting for different symmetry. **e** Spin-resolved projected density of states (PDOS) for RuO_6_ coordination polyhedron in different BERO samples.

From the viewpoint of structural stability, two typical O 2*p* orbitals (π/σ) are engaged to metal site (marked by M) for forming M-O bonds with octahedral MO_6_ atomic configuration as shown in Fig. 1a, leading to a series of (M-O) bonding levels (a_1g_/e_g_/t_2g_, with oxygen feature) and (M-O)* antibonding levels (a*_1g_/e*_g_/t*_2g_, with metal feature)^25,26^. The larger energy splitting between (M-O) and (M-O)* can lead to a higher formation energy, which is proportional to the electronegativity difference between metal site (M) and coordinated oxygen atoms. Moreover, a higher crystal symmetry makes (M-O)* antibonding level split into one empty upper band (marked by UB) and one filled lower band (marked by LB), as shown in the left panel of Fig. 1b. The half-filled electronic structure is inversely proportional to the orbital degeneracy and thus strongly relates with the structural symmetry breaking induced by coordinated O atoms. In this case, the electrons can be easily acquired from the filled lower band (LB) to increase the valence state of the metal site in acidic OER, damaging the structural stability. The lower symmetry can reduce the orbital degeneracy and rearrange the electronic occupation as shown in the middle panel of Fig. 1b, making the filled lower band (LB) into the (M-O) binding levels to hinder over-oxidation of metal site. This is because that the acquired electrons during OER have to resort to the M-O bonding levels, which benefits for enhancing structural stability but decreases catalytic activity. If we can control the symmetry breaking with particular spin polarization to reconfigure spin electron occupation as shown in the right panel of Fig. 1b, the structural stability and catalytic activity will be ensured simultaneously. The half-filled lower bands can split into spin-down ones (marked by SD-LB) that entered into the (M-O) binding levels to hinder over-oxidation and spin-up ones with metal feature (marked by SU-LB) that can provide enough valence electrons to support subsequent OER process.

To realize this concept, the crystal structure of Bi_2_Ru_2_O_7_ should be understood as shown in Fig. 1c, in which the RuO_6_ coordination can be described as a regular octahedron. At low-temperature (<150 K)^21^, the Bi_2_Ru_2_O_7_ has an O_h_ point symmetry that is trigonal antiprismatic (marked by ordered configuration), which can be attributed to the trigonal compression along three-fold symmetry axis. In addition, each Ru atoms has six equidistant Bi nearest neighbors arranged in a planar hexagon perpendicular to the three-fold axis. Under room-temperature conditions, the Bi-6*s* lone pair electrons can affect the bonding interaction between Ru and O atoms and leads to a structural disorder (marked by disordered configuration), which can be described by D_3d_ point symmetry^21^. When some temperature-insensitive Er atoms are intentionally introduced to partially replace Bi sites, the structural disorder for RuO_6_ coordination polyhedron in Bi_x_Er_2-x_Ru_2_O_7_ is topically decreased (marked by half-disordered configuration), partially limiting the symmetry transition from O_h_ to D_3d_ (named as D'_3d_ point group). As the symmetry breaking in Fig. 1d, the degenerated d_xy_, d_xz_ and d_yz_ orbitals in O_h_ symmetry split into a completely filled e_g_ band and an empty a_1g_ band for D_3d_ symmetry. Interestingly, the orbital splitting can be reduced in half-disordered structure with D'_3d_ symmetry, in which the e_g_ electrons can easily hop onto unfilled a_1g_ orbitals by electron-phonon interaction according to Goodenough-Kanamori rule^27,28^, leading to an asymmetric spin electron occupation depended on symmetry breaking.

To better understand the changes in electronic structure, the spin-resolved projected density of states (PDOS) for RuO_6_ coordination polyhedron in Bi_x_Er_2-x_Ru_2_O_7_ with different symmetry are compared in Fig. 1e. For the O_h_ symmetry, the Fermi energy (E_F_) has been completely occupied by PDOS that also occurs a slight spin polarization, because the high orbital degeneracy makes more electrons to occupy the t_2g_ orbitals. For D_3d_ symmetry, the generated orbital splitting makes electrons prefer to occupy the lower-energy e_g_ levels. Therefore, the Fermi energy becomes completely unfilled and the symmetric spin electron occupation also effectively suppress the emergence of spin polarization. When the orbital splitting energy is reduced in D'_3d_ symmetry, the electrons can occupy the e_g_ and a_1g_ orbitals more freely, leading to an observable spin polarization. Based on the Jose Gracia rules^29–31^ (detailed discussion in supplementary information), the bonding characteristic between catalysts and reactants can be reduced by the quantum spin exchange interactions (QSEI), meanwhile the rate constant for charge transfer reaction and spin-dependent electron mobility can be enhanced by magnetic potentials acting as selective gates. Inspired by above analysis, we can conclude that spin-related symmetry breaking induced by half-disordered hybridization in Bi_x_Er_2-x_Ru_2_O_7_ pyrochlores can be used as an ideal model material to simultaneously enhance the OER electrochemical stability and reactive activity in acidic electrolyte.

**Fig. 2:**
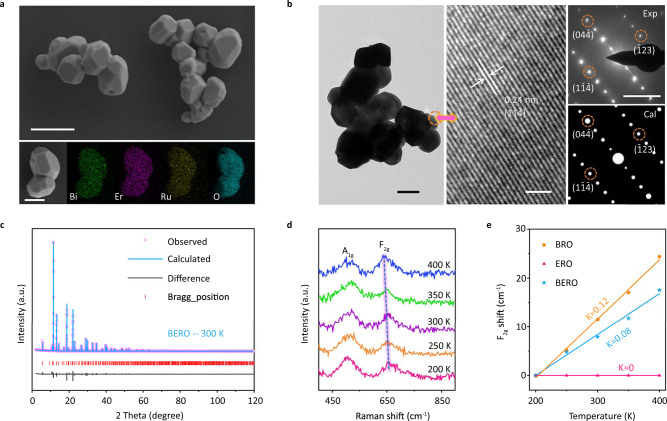
Physical characterization. **a** FE-SEM image in the upper panel (Scale bar, 500 nm) and the EDS mapping in the lower panel of the BERO (Scale bar, 200 nm) **b** TEM image in the left panel (Scale bar, 200 nm), HR-TEM image in the middle panel (Scale bar, 2 nm) and the experimental (top) and simulated (bottom) SAED pattern in the right panel (Scale bar, 5/nm) of the BERO. **c** Rietveld refinement of X-ray diffraction (XRD) patterns of as-prepared BERO. **d** Raman spectra of the BERO samples under different temperature. **e** The Raman peak shift of F_2g_ mode as a function of increasing temperature for BERO, BRO and ERO.

Subsequently, the Bi_x_Er_2-x_Ru_2_O_7_ nanoparticles were synthesized by a facile sol-gel method, which can be confirmed by low magnification EDS image (Supplementary Fig. 1). They are closely connected with each other to serve as an acidic catalyst, which can be clearly observed by scanning electron microscopy (SEM) image in Fig. 2a. The relative elemental mapping in the lower panel of Fig. 2a demonstrates that Bi, Er, Ru and O elements are uniformly distributed in these nanoparticles, displaying a ratio of 1.5:0.5:2.0:7.0 (Supplementary Fig. 1). The local regions (marked by orange circle) are enlarged to be observed by high-resolution transmission electron microscopy (HR-TEM), which demonstrates a high-crystallinity with the resolved lattice fringe of a 0.24 nm corresponding to the (1$\bar{1}\bar{4}$) plane of pyrochlores. The selected area electron diffraction (SAED) in the right panel demonstrates a D'_3d_ symmetry (marked by Exp), which also can be successfully confirmed by the simulated patterns (marked by Cal).

The final Rietveld refinement plot for synchrotron radiation X-ray diffraction (XRD) patterns are displayed in Fig. 2c and Supplementary Figs. 2–4 and Supplementary Tables 2–4, in which the refined cell parameter for Bi_1.5_Er_0.5_Ru_2_O_7_ (named BERO) with half-disordered atomic configuration (D'_3d_ symmetry) can be in good agreement with experimental observation. Interestingly, we have also tested this model with O_h_ or D_3d_ symmetry for Bi site distribution similar to that reported Bi_2_Ru_2_O_7_ (named BRO)^21^. Combined with the final Rietveld refinement results and distorted RuO_6_ structures analysis, we can conclude that atomic disordered-hybridization can be regulated by implanting particular Er atoms. Additionally, schematic illustrations of distorted RuO_6_ structures from O_h_ symmetry to D_3d_ symmetry transition have been provided in Supplementary Fig. 5 as a comparison. To further confirm the symmetric changes, the temperature-dependent in-situ Raman spectra are conducted in Fig. 2d to disclose the atomic disordering difference in symmetry breaking process. The two characteristic peaks at 504 cm^−1^ and 652 cm^−1^ can be attributed to the O-Ru-O vibrational A_1g_ mode and Bi-O-Ru vibrational F_2g_ mode^32,33^. Previous report has confirmed that the 6*s* lone pair electrons of Bi site is sensitive to electron-phonon interaction^21^, which makes the A_1g_ mode depended on atomic disordered-hybridization become more sensitive to changing temperature. To better display this point, the Raman peak shift of F_2g_ mode as a function of increasing temperature for BRO, ERO and BERO are compared in Fig. 2e. For pristine ERO sample, the Raman peak of F_2g_ mode keep unchanged as increasing temperature (Supplementary Fig. 6) because the atomic disordered-hybridization of Er site is not responsive to the thermal perturbation. Therefore, the symmetry breaking induced by atomic disordered-hybridization in pristine BRO sample can be effectively restricted by implanting some Er sites, which can be confirmed by the decreased gradient from K = 0.12 (BRO) to K = 0.08 (BERO). In a word, the structural symmetry breaking of RuO_6_ coordination polyhedron in Bi_x_Er_2-x_Ru_2_O_7_ can be triggered by atomic disorder of Bi-6*s* lone pair electrons but that can be controlled by implanting temperature-independent Er atoms, finally leading to an intriguing half-disordered hybridization.

**Fig. 3:**
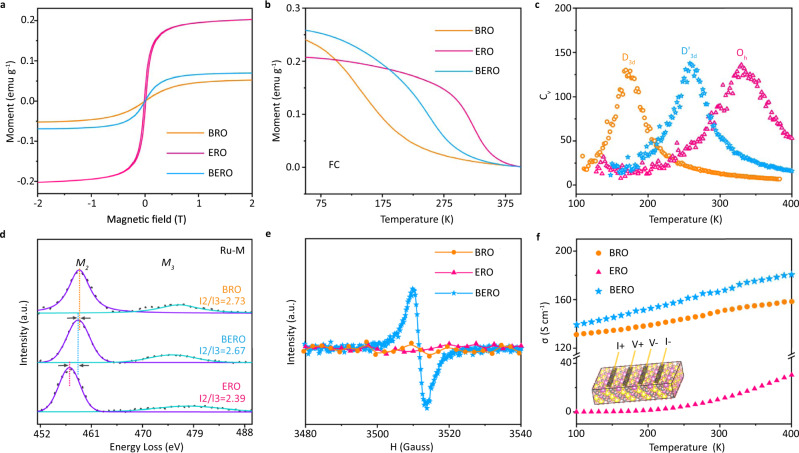
Spin-related electronic structure characterization. **a** Room temperature magnetic hysteresis loops of BERO, BRO and ERO. **b** Temperature dependence of the magnetic susceptibility measured at 1000 Oe external magnetic-field cooling (FC). **c** The specific heat *C*_*v*_ as a function of temperature. **d** EELS spectra, (**e**) ESR spectra and (**f**) The electrical conductivity of BERO, BRO and ERO.

To obtain the insight into the spin-related electronic occupation as symmetry breaking, the magnetization as a function of magnetic field (M-H) for different pyrochlores are conducted in Fig. 3a by a superconducting quantum interference device (SQUID) at room temperature. They all display nonlinear hysteresis loop curves, demonstrating a room-temperature ferromagnetic feature. It is interesting to note that the saturation magnetization intensity of ERO with ordered atomic configuration is obviously higher than disordered BRO and half-disordered BERO, which can be attributed to the symmetry-breaking-related spin electron reconfiguration. Based on the Goodenough-Kanamori rule^27,28^, the ferromagnetic feature is strongly determined by electronic hopping between completely filled e_g_ level and empty a_1g_ orbital (Supplementary Fig. 7). The analysis about XRD patterns and Raman spectra have confirmed that the atomic disordered-hybridization can be reflected by the thermal disturbance. The symmetry-breaking-induced orbital splitting plays a critical role in determining spin electron occupation and their exchange interaction. To better understand this point, the saturation magnetization intensity as a function of temperature are displayed in Fig. 3b, in which the temperature of the spin structure transition in BRO is obviously lower than ERO and BERO. This is due to the fact that the thermal-driven symmetry breaking from O_h_ to D'_3d_ or D_3d_ makes the asymmetric spin triplet occupation transform into symmetric spin singlet configuration, finally leading to a temperature-differentiated ferromagnetic transition. It is interesting to note that a higher ferromagnetic feature plays an important role in enhancing OER performance (Supplementary Figs. 8–10).

To disclose the physical mechanism, Monte Carlo simulation using a 13 × 13 × 13 spin matrix is constructed to investigate the spin structure transformation. The spin-related Hamiltonian can be described as: H = (J_1_ + J_2_ + J_3_) **S**_**i**_·**S**_**j**_, in which **S**_**i**_ and **S**_**j**_ are the spin net, respectively. The J_1_, J_2_ and J_3_ corresponds to the first-, second-, and third-nearest spin coupling strength, which can be calculated by symmetry-breaking-related spin electronic structure based on the Heisenberg model^27,34^. For a given temperature, the specific heat with spin flip can be calculated as ${C}_{v}=(\langle {E}^{2}\rangle -{\langle E\rangle }^{2})/{K}_{B}{T}^{2}$, where *E* is the total energy of the system. Then, the specific heat C_v_ as a function of temperature for different symmetry structure is calculated in Fig. 3c. It can be observed that the spin flip temperature at high-symmetric O_h_ configuration is obviously higher than D_3d_ and D'_3d_ configuration. This comparison further demonstrates that the spin electron configuration and exchange interaction is strongly related with orbital splitting induced by symmetry transition.

Subsequently, the electron energy loss spectroscopy (EELS) measurements of Ru M-edge^35,36^ are conducted in Fig. 3d to disclose the symmetry-breaking-induced spin-resolved electronic structure transformation. It is universally accepted that the changes in Ru-M edge are strongly depended on the crystal symmetry and spin electron configuration, in which a higher relative integrated intensity (I_2_/I_3_) between M_2_ and M_3_ peaks represent a lower symmetry and spin electron occupation. Compared to the high symmetric ERO sample, the Ru M_2_ peaks in BRO and BERO are shifted to high-energy region meanwhile the relative intensities I_2_/I_3_ are also enhanced correspondingly, which can be ascribed to the contribution of multistage orbital hybridization. The symmetry breaking can generate a series of nondegenerate orbitals that makes partial e_g_ electrons easily hop onto a_1g_ levels more easily, leading to an asymmetric spin electron occupation. To identify the influence of Bi-6*s* lone pair electrons onto the structural transition, electron spin resonance (ESR) spectra for different samples are provided in Fig. 3e. Compared to BRO, the ESR intensity is obviously increased in BERO because the introduced Er atoms can effectively protect the Bi-6*s* lone pair electrons. That is why the symmetry breaking can be partially hindered in BERO.

To understand the correlation between thermal disturbance and symmetry breaking induced by atomic disorder, the temperature-dependent conductivity (σ) is displayed in Fig. 3f to reflect the electron-phonon interaction. Interestingly, the value of pristine BRO is obviously higher than that of ERO, which inspires us to conclude that the enhanced conductivity in BERO only can be attributed to the multistate orbital hybridization induced by symmetry breaking. More importantly, the conductivity difference between BERO and BRO is enlarged as increasing temperature. In principle, atomic displacement in sample without symmetry breaking is more localized and cannot be affected by high-energy hot carrier, which causes a single scattering electron-phonon interaction. When symmetry breaking is triggered by atomic disordered-hybridization, the atomic displacement becomes delocalized that can lead to multiple electron-phonon scattering. With increasing temperature, the atomic disordered-hybridization can be effectively hindered by implanting Er atoms, which makes the multiple electron-phonon scattering transform into single scattering event. The changes in hot carrier scattering are responsible for their conductivity difference.

### Practical application and catalytic mechanism of OER

**Fig. 4:**
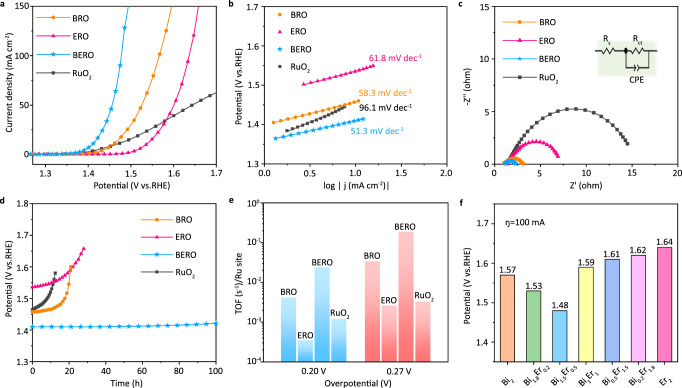
Practical application of OER. **a** Polarization curves and (**b**) Tafel plots of the different samples. **c** EIS curves (Inset: the equivalent circuit.) and (**d**) Stability test of BERO, BRO, ERO and commercial RuO_2_. **e** TOF results at overpotential of 0.2 V and 0.27 V. **f** The results of the potential at 100 mA cm^−2^ for Bi_x_Er_2-x_Ru_2_O_7_ with different ratios.

For a fair comparison in OER performance, the normalizations of the currents based on active surface area (ECSA), surface areas (BET method) and corresponding mass activity, specific activity are provided in Supplementary Figs. 11–13. The compared results confirm that the OER performance for BERO sample is obviously better than BRO and ERO, which are independent of normalization methods. To better understand the contribution of spin-related symmetry breaking, the linear sweep voltammetry curves of the catalysts with different symmetry are provided in Fig. 4a. It is interesting to note that the BERO sample with half-disordered atomic configuration is completely superior to the pristine BRO, ERO and commercial RuO_2_ catalysts especially at high current density. This experimental conclusion is quite agreement with aforementioned octahedral crystal field theory in Fig. 1 that the asymmetric spin electron occupation induced by half-disordered atomic configuration plays a critical role in improving the OER catalytic activity. Based on the QSEI description, the selectivity of reaction pathway is also related with the electronic occupation at d orbital^29–31^, as shown in Supplementary Fig. 9. For metal active site, the catalytic activity is strongly depended on the combination of empty and occupied a_1g_ and e_g_ orbitals. To realize the reactant chemisorption, the metal sites need to an empty a_1g_ orbitals to accept the valance electrons of reactants. On the contrary, to reduce the activation barrier at OER, the metal site must possess some separate d electrons that can donate into the antibonding orbital to weak the orbital interaction with intermediates. Therefore, “acceptance-donation” of electrons is the essential interaction between the metal site and reactants, where the regulation of empty and occupied d orbitals is responsible for the enhanced catalytic activity. In this regard, the Ru sites with half-filled a_1g_ orbital can be used to accept the valence electrons of intermediates that can donate into the bonding orbital (σ and π) to accelerate the OER process. In the high-symmetric ERO sample, the Ru-4*d* orbitals with high catalytic activity have been entered into Ru-O bonding level of RuO_6_ coordination polyhedron, which cannot support good catalytic activity. On the contrary, the disordered atomic configuration in pristine BRO makes the degenerated t_2g_ orbital split into completely filled e_g_ and empty a_1g_ orbitals, in which the Ru sites cannot easily provide unpaired valance electrons to bond with the reactants. As the structural transition from D_3d_ (BRO) to D'_3d_ (BERO), the degenerated d_xy_, d_xz_ and d_yz_ orbitals in D'_3d_ point group split into a half-filled e_g_ band and a_1g_ band, which are advantages to accelerate “acceptance-donation” process. That is why the optimal catalytic activity occurs at BERO sample.

To further disclose their difference in catalytic activity, the corresponding Tafel slopes are displayed in Fig. 4b. Compared to pristine BRO (58.3 mv dec^−1^) and ERO (61.8 mv dec^−1^), the Tafel slope is decreased to 51.3 mv dec^−1^ in BERO with controlling symmetry breaking, demonstrating a possible transformation in the rate-limiting barrier because of disorder-dependent orbital interaction and charge transfer between the active sites and reactants. Additionally, the relative electrochemical impedance spectra (EIS) in Fig. 4c also confirms that the carrier transfers are also strongly depended on the spin-related symmetry breaking. Compared to the behavior of ERO, ERO and commercial RuO_2_ catalysts, the charge transfer resistance (R_ct_) can be sharply decreased in BERO, demonstrating that the half-disordered orbital degeneracy not only displays a higher reactive activity but also can accelerate the carrier transfer^37,38^. This is because that the electron transfer ability is also related with the magnetic structure transition induced by this particular spin-reconfiguration (Supplementary Figs. 8–10). More importantly, the electrochemical stability of BERO sample at ~10 mA cm^−2^ current can be substantially enhanced comparing to pristine BRO, ERO and RuO_2_, as shown in Fig. 4d. After 100 h OER test in acidic electrolyte, no obvious potential decay in BERO can be observed. The original morphology, crystal symmetry and chemical composition cannot be affected by the long-time acidic OER stability testing (Supplementary Figs. 14–18 and Supplementary Tables 6–12).

According to the oxygen production and the number of active sites, the turnover frequency (TOF) of different samples are calculated^39,40^ and compared in Fig. 4e. The BERO sample displays the highest TOF value with 0.024 s^−1^ at 0.20 V overpotential, which is obviously higher than BRO (0.0042 s^−1^) and ERO (0.00035 s^−1^). When the overpotential is increased to 0.27 V, the TOF values are increased slightly but the general feature keeps unchanged. This catalytic activity difference also can be confirmed by electrochemically active surface area (ECSA) in Supplementary Fig. 19. This comparison implies that the activation barrier of OER at BERO surface should be smaller than the others, which makes the intermediates become easier to be adsorbed and dissociated from active site. This difference in reaction rate only can be attributed to the disorder-hybridized electronic structure.

Finally, the catalytic activity of various Bi_x_Er_2-x_Ru_2_O_7_ with different disordered-hybridization are compared in Fig. 4f (the detailed experiments in Fig. 4a and Supplementary Fig. 20). The optimal OER performance occurs at Bi_1.5_Er_0.5_Ru_2_O_7_, which is obviously more excellent than the other samples. This is because that orbital degeneracy of RuO_6_ coordination polyhedron in Bi_x_Er_2-x_Ru_2_O_7_ can be regulated by doping Er concentration, thus leading to a changeable catalytic activity. For x = 1.5, non-equivalent atomic occupation of Er site can partially eliminate the disordered-hybridization induced by Bi-6*s* lone pair electrons, which can lead to a suitable multistate electronic structure to trigger spin reconfiguration. That is why we choose this sample as an ideal candidate. In addition, the comprehensive performances of BERO are collected to compare with current reports in Supplementary Table 13, demonstrating an obvious advantage in OER application. To confirm the universal feasibility of atomic half-disordering method, the OER performances for Pb_x_Er_2-x_Ru_2_O_7_ are also conducted in Supplementary Fig. 21, which indicates that the doped Er element also can enhance the OER performance in the Pb_x_Er_2-x_Ru_2_O_7_, similar to that of BERO.

**Fig. 5:**
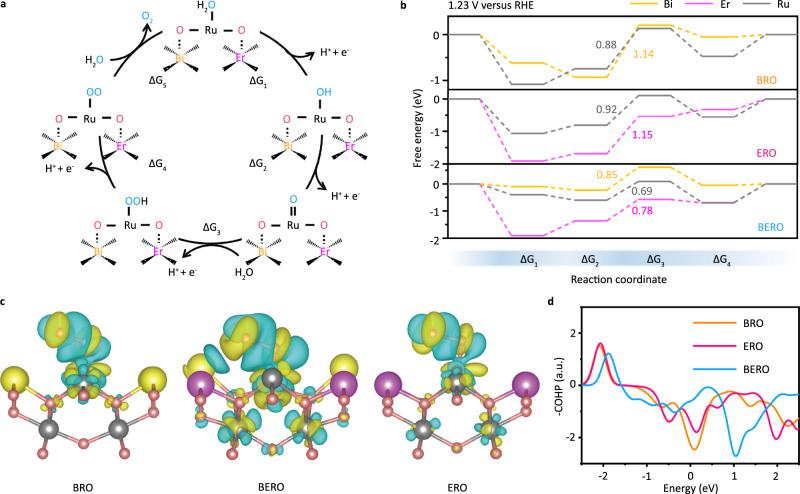
Correlation of OER mechanism with different disordered-hybridization. **a** The OER process of BERO. **b** Free energies of OER steps at different reactive sites. **c** The charge density difference of adsorbed *OOH onto RuO_6_ coordination polyhedron in BERO samples with different symmetry breaking. **d** The crystal orbital Hamilton population (COHP) of adsorbed *OOH onto Ru site.

To reveal the correlation between disordered atomic configuration and fundamental catalytic mechanism, the OER pathway composed of one thermodynamic process (ΔG5) and four electrochemical steps^41^ are demonstrated in Fig. 5a, with the Ru-*OH formation as a starting point (ΔG1). To disclose the contribution of atomic half-disorder onto acidic oxygen evolution, the key differences for different disordered BERO pyrochlores with spin-related symmetry breaking can be considered as the structural distortion of RuO_6_ coordination polyhedron and then to act as active sites. The crystal structures of BRO, ERO and BERO with D_3d_, O_h_, and D'_3d_ symmetry are obtained from the Rietveld refinement of XRD patterns. Therefore, the free energy differences (ΔG) between two intermediates can be used to evaluate the rate-limiting barrier and reaction pathway, as shown in Fig. 5b. For the pristine BRO with D_3d_ symmetry, the optimal active sites occur at Ru site and the formation of Ru-*OOH in step 3 can act as the rate-limiting step with a 0.88 eV potential barrier, which is obviously lower in energy than that of Bi-*OOH (1.14 eV). For the ERO with O_h_ symmetry, the acidic OER still prefer to occur at Ru site and the potential-determining intermediates also happens at step 3. In this case, the formation of *OOH at Er site needs more energy (1.15 eV) to be realized, which is obviously higher than the corresponding intermediates at Ru site (0.92 eV). We can conclude that the acidic OER is authentically depended on the symmetry of RuO_6_ coordination polyhedron.

When the symmetry breaking is considered into BERO with D'_3d_ symmetry, the rate-limiting activation energies at Ru, Bi and Er sites are markedly lower than the corresponding active site in BRO and ERO. More importantly, the free energy differences of rate-limiting reactants among these metal sites are decreased due to spin electron asymmetric occupation and multiage orbital hybridization. For example, the potential-determining step is determined by the formation of *OOH and the relative action barrier on Ru, Bi and Er sites are reduced to 0.69 eV, 0.85 eV and 0.78 eV, respectively. Based on the lowest principle of energy, the catalytic activity can be enhanced by this half-disordered hybridization but the OER more prefer to occur at Ru site, which is strongly related with atomic half-disordered hybridization. The bonding interactions between catalysts and reactants are also related with this change in electronic reoccupation (Supplementary Fig. 22).

To understand the spin-related orbital interaction and charge transfer between active sites and the rate-limiting reactants, the charge density differences of adsorbed *OOH onto RuO_6_ coordination polyhedron with different symmetry breaking are calculated and shown in (Fig. 5c). The yellow isosurface corresponds to the electron depletion region and the blue one represents the electron increase zone. When the rate-limiting intermediate of *OOH is formed onto Ru site, the unpaired O-2*p* orbitals prefer to hybridize with Ru-4*d* orbital. Compared to the pristine BRO and ERO surface, the electron decrease zones become more delocalized at the coordinated Ru-OOH bonds, which makes the t_2g_ electron occupation of Ru-4*d* orbital decrease to 3.783 (BERO) from 3.804 (BRO) and 3.806 (ERO). More interestingly, the spin magnetic moment of BERO is simultaneously decreased to 0.166 μ_B_ from 1.994 μ_B_ (BRO) and 1.195 μ_B_ (ERO), demonstrating the OER process is also strongly related with spin-related symmetry breaking.

The bonding interaction between active sites and reactants generally demonstrate a mixed ionic-covalent feature due to the energetic similarity and spatial wave function overlapping between Ru-4*d* orbital and O-2*p* orbitals, which plays a primary role in determining reactive activity. To better understand this point, the crystal orbital Hamilton population (COHP) is compared in Fig. 5d to disclose the bonding characteristic of active sites with different symmetry breaking. Different from the Ru-*OOH antibonding contribution in BRO and ERO, an obvious bonding states appear at Fermi level in BERO, demonstrating that the valence electrons of Ru-4*d* orbital can partially transfer to the half-filled O-2*p* orbitals more easily. The relative stability difference of Ru-*OOH bond in BERO is weaker than that of ERO and BRO, thus leading to a lower activation barrier.

## Discussion

In this work, we suggested that the spin-related symmetry breaking induced by half-disordered hybridization in Bi_x_Er_2-x_Ru_2_O_7_ to enhance the electrochemical stability and reactive activity, through designing the orbital degeneracy and spin electron occupation. As a result, the charge transfer and orbital interaction between the active sites and intermediates demonstrate a spin-reconfiguration-dependent catalytic kinetics, making the electrodes work at super-low overpotential ~0.18 V at 10 mA cm^−2^ accompanied with excellent stability of 100 h in acidic electrolyte. Our findings open a new door to enhance the acidic OER performance in the fields of symmetry-breaking-driven spin-related electrocatalysis.

## Methods

### Preparation of BERO electrode

All chemicals were of analytical grade and used as received without further purification. The BERO pyrochlore oxide nanoparticles were synthesized by a facile sol-gel route by using citric acid as chelating agent. In a typical process, Bi(NO_3_)_3_·5H_2_O (0.364 g), ErCl_3_·6H_2_O (0.095 g), ruthenium (III) nitrosyl nitrate (6.180 mL) and citric acid (0.840 g) were dispersed in deionized water under continuously stirring at room temperature for 1 h. Then, 340 μL of perchloric acid was injected into the above solution using a pipette at a rate of 0.1 mL min^−1^ and the mixture was stirred under the high-purity argon (Ar 99.999%) current protection for 30 min to form a homogeneous solution at room temperature. Thereafter, the precursor solution was transferred to rotary evaporation at 85 °C under stirring at 900 rpm and then obtained viscous gel. Subsequently, the transparent gel was dried in a vacuum oven at 125 °C for 12.5 h to dehydrate and then heated in the furnace at 500 °C for 4 h in air to form a solid precursor. Finally, the above precursor after ball milling was calcined at 1000 °C with a rate of 5 °C per minute heating up for 8 h in air to form BERO powder. With the exception of the different raw materials, the other catalysts (e.g., BRO, and ERO) were successfully prepared by the similar combustion procedure as BERO.

### Electrochemical OER measurements

All of the electrochemical measurements in this work were tested in O_2_-saturated 0.1 M HClO_4_ solution using a standard three-electrode electrochemical cell equipped with an electrochemistry workstation (Ivium Vertex.C.EIS). A standard three-electrode electrolyte was used in all tests, with a graphite rod (Alfa Aesar, 99.9995%) as the counter electrode, commercial Ag/AgCl (saturated KCl) as the reference electrode, and carbon paper loaded with different samples as the working electrodes. For a comparison, commercial RuO_2_ powders were loaded on carbon paper via drop-casting of a catalyst ink. The homogeneous catalyst ink was prepared by mixture of 800 μL ethanol, 300 μL deionized water, 10 mg of catalyst powder and 100 μL Nafion solution (5 wt.%, Du Pont). Before the OER kinetics measurements, the electrolyte was bubbled with high-purity oxygen for at least 20 min to ensure the H_2_O/O_2_ equilibrium. All the data were presented with iR correction through reversible hydrogen electrode (RHE) calibration. The linear sweep voltammetry (LSV) was recorded with the scan rates of 5 mV s^−1^. The external potentials [E(Ag/AgCl)] were measured against the Ag/AgCl electrode as reference which could convert to the potential versus RHE by using the Nernst function: ${{{{{\rm{E}}}}}}\left({{{{{\rm{RHE}}}}}}\right)=E\left({Ag}/{AgCl}\right)+{E}^{0}({Ag}/{AgCl})+0.059{{{{{\rm{PH}}}}}}$, where ${E}^{0}\left({Ag}/{AgCl}\right)$ is the standard electrode potential of Ag/AgCl reference electrode. The Tafel plots was obtained by converting the J-V curves as potential (V vs. RHE) versus the logarithm of current density (log |J|). The stability of the catalysts was reflected by the chronopotentiometry measurements, which were tested by maintaining the current density of 10 mA cm^−2^ for 100 h. The electrochemical impedance spectra (EIS) were recorded under the following conditions: AC potential perturbation 5 mV, frequency ranges 100 kHZ to 0.01 Hz, and open circuit. To determine the double-layer capacitance (C_dl_), the cyclic voltammograms were tested at various scan rates (10–100 mV s^−1^) between 1.20 V and 1.30 V (vs. RHE)^42,43^.

### Density functional theory (DFT) calculation

The theoretical calculations are acquired from Vienna ab initio simulation package (VASP), with Perdew-Burke-Ernzerhof (PBE) and generalized gradient approximation (GGA)^44,45^. The Monkhorst-Pack k-points grid of 9 × 9 × 1 and the cutoff energy of 460 eV are adopted to expand the Kohn-Sham wave functions. Additionally, the vacuum space of 20 Å is used into slabs, which can be used to exclude the interaction between periodical images. In our calculation, the atomic forces were converged to be 0.01 eV/Å.

### Reporting summary

Further information on research design is available in the Nature Research Reporting Summary linked to this article.

## Supplementary information

Supplementary Information

Reporting Summary

## Data Availability

The data described in this paper are available from the authors upon reasonable request.

## References

[1] Suen NT (2017). Electrocatalysis for the oxygen evolution reaction: recent development and future perspectives. Chem. Soc. Rev..

[2] An L (2021). Recent development of oxygen evolution electrocatalysts in acidic environment. Adv. Mater..

[3] Retuerto M (2019). Na-doped ruthenium perovskite electrocatalysts with improved oxygen evolution activity and durability in acidic media. Nat. Commun..

[4] Chen YB (2019). Exceptionally active iridium evolved from a pseudo-cubic perovskite for oxygen evolution in acid. Nat. Commun..

[5] Huang JZ (2021). Modifying redox properties and local bonding of Co_3_O_4_ by CeO_2_ enhances oxygen evolution catalysis in acid. Nat. Commun..

[6] Wu J (2019). A general synthesis approach for amorphous noble metal nanosheets. Nat. Commun..

[7] Zhuang ZW (2019). Three-dimensional open nano-netcage electrocatalysts for efficient pH-universal overall water splitting. Nat. Commun..

[8] Miao XB (2019). Quadruple perovskite ruthenate as a highly efficient catalyst for acidic water oxidation. Nat. Commun..

[9] Montiel M, Hernández-Fernández P, Fierro JLG, Rojas S, Ocón P (2009). Promotional effect of upper Ru oxides as methanol tolerant electrocatalyst for the oxygen reduction reaction. J. Power Sources.

[10] Kim BJ (2017). Unraveling thermodynamics, stability, and oxygen evolution activity of strontium ruthenium perovskite oxide. ACS Catal..

[11] Kim J (2018). A porous pyrochlore Y_2_[Ru_1.6_Y_0.4_]O_7-δ_ electrocatalyst for enhanced performance towards the oxygen evolution reaction in acidic media. Angew. Chem. Int. Ed..

[12] Kim M (2019). Reducing the barrier energy of self-reconstruction for anchored cobalt nanoparticles as highly active oxygen evolution electrocatalyst. Adv. Mater..

[13] Hubert MA (2020). Acidic oxygen evolution reaction activity-stability relationships in Ru-based pyrochlores. ACS Catal..

[14] Feng Q (2020). Influence of surface oxygen vacancies and ruthenium valence state on the catalysis of pyrochlore oxides. ACS Appl. Mater. Interfaces.

[15] Kim M, Park J, Kang M, Kim JY, Lee SW (2020). Toward efficient electrocatalytic oxygen evolution: emerging opportunities with metallic pyrochlore oxides for electrocatalysts and conductive supports. ACS Cent. Sci..

[16] Pittkowski RK (2021). Synergistic effects in oxygen evolution activity of mixed iridium-ruthenium pyrochlores. Electrochim. Acta.

[17] Kim J (2017). High-performance pyrochlore-type yttrium ruthenate electrocatalyst for oxygen evolution reaction in acidic media. J. Am. Chem. Soc..

[18] Lebedev D (2017). Highly active and stable iridium pyrochlores for oxygen evolution reaction. Chem. Mater..

[19] Sardar K (2014). Water-splitting electrocatalysis in acid conditions using ruthenate-iridate pyrochlores. Angew. Chem. Int. Ed..

[20] Sun YM (2020). Spin-related electron transfer and orbital interactions in oxygen electrocatalysis. Adv. Mater..

[21] Avdeev M, Haas MK, Jorgensen JD, Cava RJ (2002). Static disorder from lone-pair electrons in Bi_2-x_M_x_Ru_2_O_7-y_ (M = Cu, Co; x = 0, 0:4) pyrochlores. J. Solid State Chem..

[22] Radosavljevic I, Evans J, Sleight A (1998). Synthesis and structure of pyrochlore-type bismuth titanate. J. Solid State Chem..

[23] Gardner JS, Ehlers G (2009). Magnetic order and crystal field excitations in Er_2_Ru_2_O_7_: a neutron scattering study. J. Phys.: Condens. Matter.

[24] Tachibana M (2006). Electronic properties of the metallic pyrochlore ruthenates Pb_2_Ru_2_O_6.5_ and Bi_2_Ru_2_O_7_. Phys. Rev. B.

[25] Seo DH (2016). The structural and chemical origin of the oxygen redox activity in layered and cation-disordered Li-excess cathode materials. Nat. Chem..

[26] Assat G, Tarascon JM (2018). Fundamental understanding and practical challenges of anionic redox activity in Li-ion batteries. Nat. Energy.

[27] Zhou G (2021). Spin-state reconfiguration induced by alternating magnetic field for efficient oxygen evolution reaction. Nat. Commun..

[28] Goodenough J (2008). Goodenough-Kanamori rule. Scholarpedia.

[29] Gracia J, Sharpe R, Munarriz J (2018). Principles determining the activity of magnetic oxides for electron transfer reactions. J. Catal..

[30] Gracia J (2017). Spin dependent interactions catalyse the oxygen electrochemistry. Phys. Chem. Chem. Phys..

[31] Biz C, Fianchini M, Gracia J (2021). Strongly correlated electrons in catalysis: focus on quantum exchange. ACS Catal..

[32] Feng XH (2021). Unraveling the principles of lattice disorder degree of Bi_2_B_2_O_7_ (B = Sn, Ti, Zr) compounds on activating gas phase O_2_ for soot combustion. ACS Catal..

[33] Zhang XH (2017). Ni/Ln_2_Zr_2_O_7_ (Ln = La, Pr, Sm and Y) catalysts for methane steam reforming: on the effects of A site replacement. Catal. Sci. Technol..

[34] Dev P, Xue Y, Zhang PH (2008). Defect-induced intrinsic magnetism in widegap III nitrides. Phys. Rev. Lett..

[35] Laïk B, Bourg S, Pereira-Ramos JP, Bruyère S, Pierson JF (2015). Electrochemical reaction of lithium with ruthenium nitride thin films prepared by pulsed-DC magnetron sputtering. Electrochim. Acta.

[36] Scofield ME (2016). Correlating the chemical composition and size of various metal oxide substrates with the catalytic activity and stability of as-deposited Pt nanoparticles for the methanol oxidation reaction. Catal. Sci. Technol..

[37] Xu ZZ (2022). Electronic reconfiguration induced by neighboring exchange interaction at double perovskite oxide interface for highly efficient oxygen evolution reaction. Chem. Eng. J..

[38] Zhou G (2019). Photoinduced semiconductor-metal transition in ultrathin troilite FeS nanosheets to trigger efficient hydrogen evolution. Nat. Commun..

[39] Lin YC (2019). Chromium-ruthenium oxide solid solution electrocatalyst for highly efficient oxygen evolution reaction in acidic media. Nat. Commun..

[40] Ng JWD (2016). Gold-supported cerium-doped NiO_x_ catalysts for water oxidation. Nat. Energy.

[41] Huang ZF (2019). Chemical and structural origin of lattice oxygen oxidation in Co-Zn oxyhydroxide oxygen evolution electrocatalysts. Nat. Energy.

[42] Fu Y (2019). Electric strain in dual metal Janus nanosheets induces structural phase transition for efficient hydrogen evolution. Joule.

[43] Fu Y (2020). Dual-metal-driven selective pathway of nitrogen reduction in orderly atomic-hybridized Re_2_MnS_6_ ultrathin nanosheets. Nano Lett..

[44] Perdew JP, Burke K, Ernzerhof M (1996). Generalized gradient approximation made simple. Phys. Rev. Lett..

[45] Zhou G (2021). Recharged catalyst with memristive nitrogen reduction activity through learning networks of spiking neurons. J. Am. Chem. Soc..

