# Supplementary material for: Spin-related symmetry breaking induced by half-disordered hybridization in BixEr2-xRu2O7 pyrochlores for acidic oxygen evolution
Source: Nat Commun. 2022 Jul 15;13:4106. doi: 10.1038/s41467-022-31874-4 (PMC9287408; doi:10.1038/s41467-022-31874-4)
Supplement: Supplementary file 1 — Supplementary Information [file 41467_2022_31874_MOESM1_ESM.pdf]

Supplementary Information

Spin-related symmetry breaking induced by half-disordered hybridization in $Bi_xEr_{2-x}Ru_2O_7$ pyrochlores for acidic oxygen evolution

Gang Zhou[1], Peifang Wang[1,*], Bin Hu[1], Xinyue Shen[2], Chongchong Liu[1], Weixiang Tao[1], Peilin Huang[1], Lizhe Liu[3,4*]

[1] *Key Laboratory of Integrated Regulation and Resource Development on Shallow Lake of Ministry of Education, College of Environment, Hohai University, Nanjing 210098, People's Republic of China*

[2]*College of Electronic and Optical Engineering, Nanjing University of Posts and Telecommunications, Nanjing 210023, People's Republic of China*

[3] *Jiangsu Key Laboratory for Nanotechnology and Collaborative Innovation Center of Advanced Microstructures, National Laboratory of Solid State Microstructures, Nanjing University, Nanjing 210093, People's Republic of China*

[4]*Guangxi Key Laboratory of Nuclear Physics and Nuclear Technology, Guangxi Normal University, People's Republic of China*

[*]e-mail: pfwang2005@hhu.edu.cn (P.F.W); lzliu@nju.edu.cn (L.Z.L)

The catalytic mechanism based on Jose Gracia rules.

The OER performance is strongly related with spin-dependent orbital hybridization in catalysts, in which the number of unpaired electrons during OER cannot be conserved. In OER process, the magnetic potentials via exchange interaction or spin-orbit coupling effect can directly affect the activation energy at rate-limiting step and carrier transfer ability. This is because that the bonding characteristic between catalysts and reactants can be reduced by the quantum spin exchange interactions, meanwhile the rate constant for charge transfer reaction and spin-dependent electron mobility can be enhanced by magnetic potentials acting as selective gates.

To understand the correlation between ferromagnetic (FM) configuration and catalytic activity, we should extend the spin-dependent mechanisms of electron tunneling to catalytic surface or interfaces, because the exchange coupling between different orbitals, in the catalysts and with the chemisorbed reactants, affects the kinetics of electron transfer reactions. In this regard, the Jose Gracia rules can be introduced to explain our experimental conclusions. To sum up, the Jose Gracia rules are as follows[1-3] : (1) The spin angular momentum is conserved during an electron transfer in the catalyst and with the reactants; (2) The intra-atomic and inter-atomic exchange interaction in the covalent framework are ferromagnetic in oxides with minimum ΔG; (3) In the active metal atoms on the surface, the d-orbitals oriented towards the bonds with the reactants, anti-bonding d-orbitals at the fermi level, must be partially occupied; (4) The overall reduction of Coulomb interactions can facilitate the entrance of itinerant charge carriers at working conditions. Defects or dopants at the interstitial sites or cations with occupied f- or d- orbitals, increasing the covalence of

frameworks, help to create active FM orderings, adaptable to redox variations and with minimum ΔH; (5) At the transition state, the active cations on the surface at reaction conditions formally receive or lose electronic density during spin-selective steps with the reactants. Nevertheless, good electrocatalysts retain the overall FM exchange delocalization; (6) The reaction mechanism adapts to the response of the electronic free energy of activation, ΔG, towards maximum entropic gains.

According to these rules, it can be found that good catalysts need to be FM feature. Generally speaking, the intra-atomic exchange interactions benefits for the configurations with more unpaired d electrons, leading to an appearance of high spin state. However, in crystal catalysts, the oriented metals and ligands are bonded together depending on the symmetry. The crystal field theory discloses that disordered-hybridization for $Bi_2Ru_2O_7$ at room temperature makes the d manifolds split into completely filled e_g orbital and empty a_{1g} orbital, the latter one is higher in energy due to the larger electronic repulsions. Interestingly, the influence of spin-orbit coupling in $Bi_xEr_{2-x}Ru_2O_7$ sample can contribute to trigger a magnetic splitting from d shells meanwhile the RuO_6 coordination are changed into D'_{3d} symmetry from D_{3d} point group. In these hybridized structures, the intra-atomic exchange interactions and crystal filed contribution becomes comparable strength, leading to a competing spin state. When the doped Er atoms lead to an uneven occupation at d shells as shown in Figure 1d, the electron-spin (e-spin) becomes localization by Jahan-Teller distortion. The electronic repulsions between the oxygen and metal orbitals decrease with the radial compression of the d orbitals that increase the intra-atomic exchange interaction but weak the crystal

field effect, thus leading to a high-spin configuration as the magnetic characterization in Figure 3a-3b. Therefore, we can conclude that the magnetic existence is originated from the cooperative phenomenon influenced by inter-atomic exchange interactions and crystal field, which plays a decisive role in electro-catalysis because they can set the collective e-spin transport. Enough empty d orbitals at the Fermi level for $Bi_xEr_{2-x}Ru_2O_7$ sample with D'_{3d} point group can enforce the exchange of electrons to generate a ferromagnetism, which benefits for a fast and coherent e-spin transport with a zero band gap for the spin-up state. Instead, if the orbitals are symmetrically filled, the intra-atomic interactions are antiferromagnetic configuration for D_{3d} symmetry with a band gap, which can be confirmed by the difference in spin-resolved DOS as shown in Figure 1e.

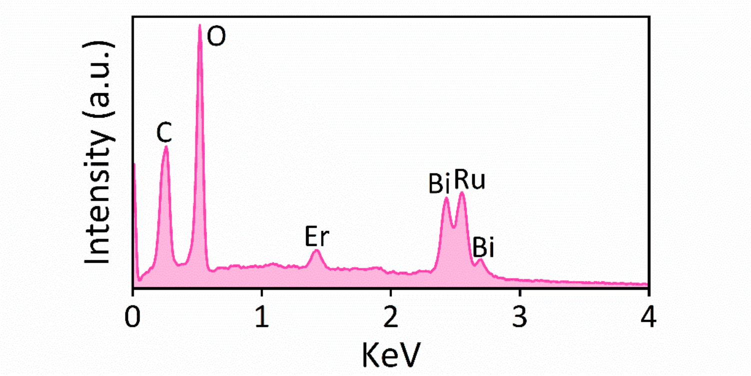

Supplementary Figure 1. EDS spectrum of the BERO nanoparticles.

No noticeable impurities are introduced unintentionally in the preparation process. The detected C element is derived from the CFC substrate. In our EDX experiments, the results are obtained under the vacuum condition and the additional influence from other oxygen species have been excluded. To obtain more credible results, the samples are measured for many times and get an average ratio. Combing with ICP method (Supplementary Table 1) and Rietveld refinement results (Supplementary Tables 2-4), the final conclusion about Bi, Er, Ru and O elements radio is about 1.5 ± 0.02, 0.5 ± 0.01, 2 ± 0.02 and 7 ± 0.04.

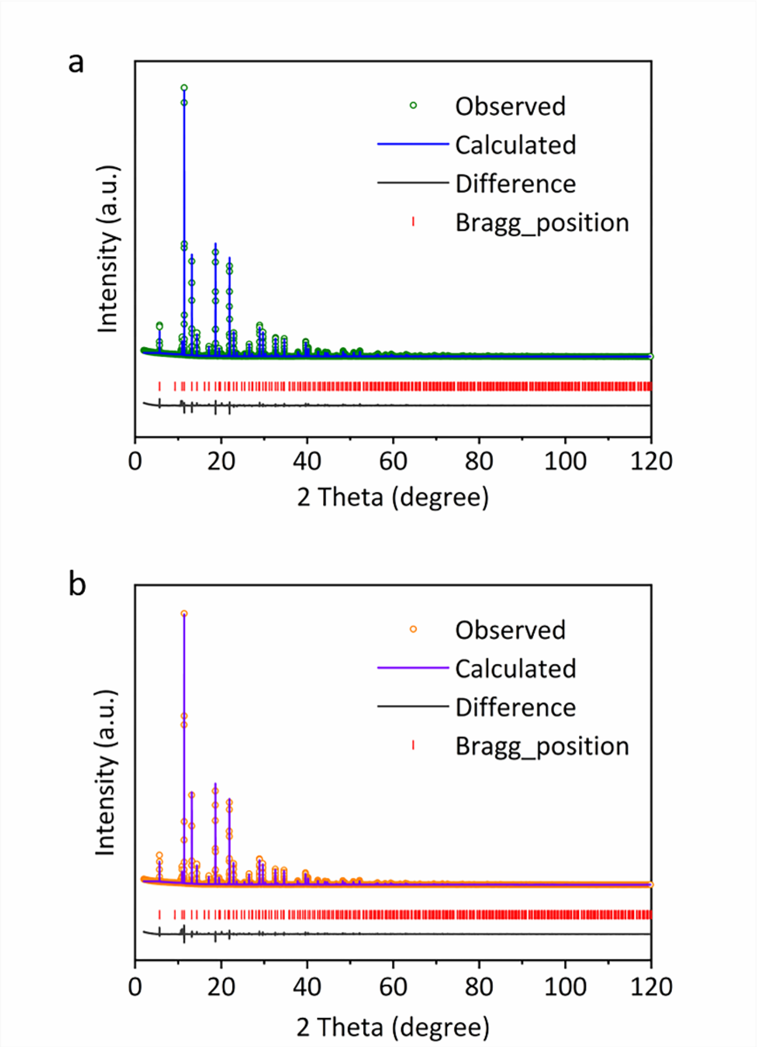

Supplementary Figure 2. Rietveld refinement of synchrotron X-ray diffraction patterns of as-prepared BRO at (a) 50K and (b) 300K. The wavelength used was 0.58889 Å.

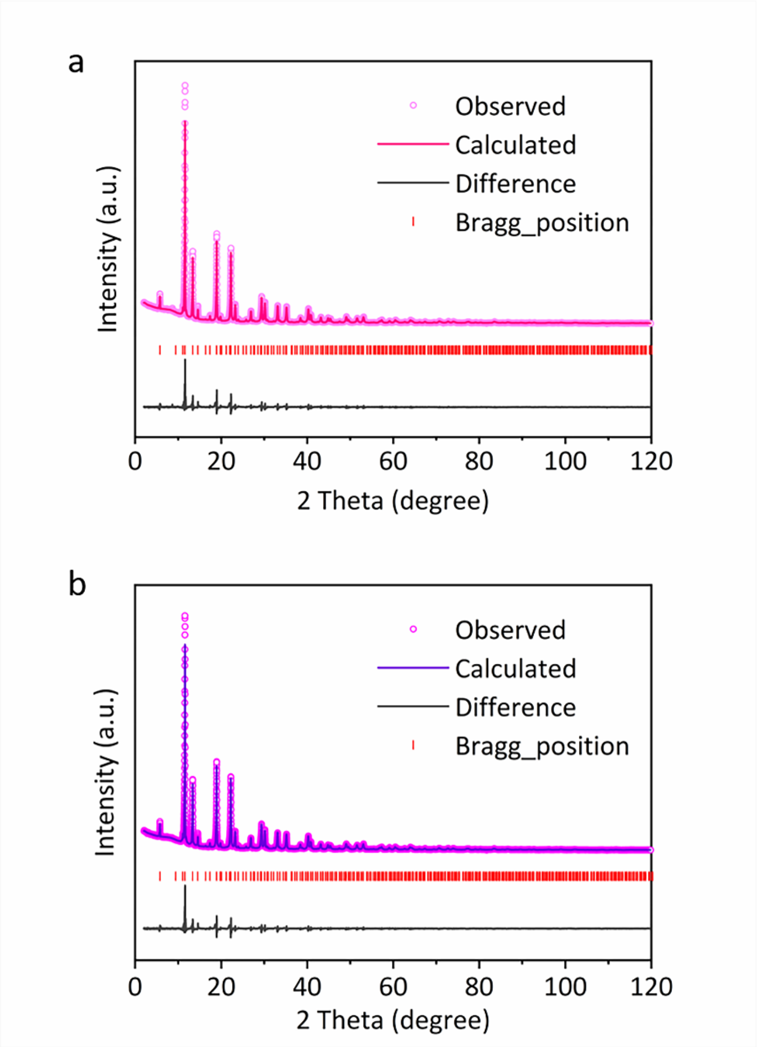

Supplementary Figure 3. Rietveld refinement of synchrotron X-ray diffraction patterns of as-prepared ERO at (a) 50K and (b) 300K. The wavelength used was 0.58889 Å.

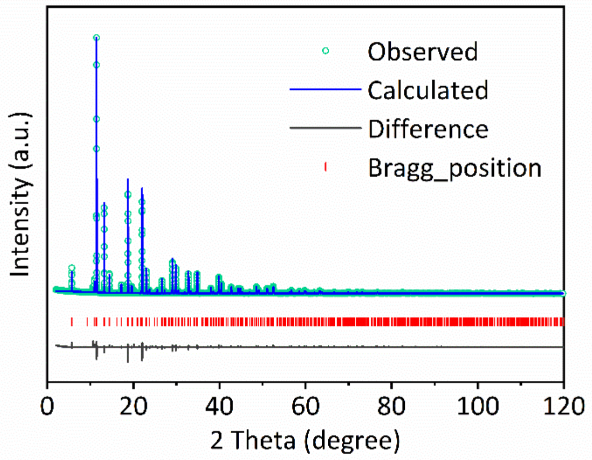

Supplementary Figure 4. Rietveld refinement of synchrotron X-ray diffraction patterns of as-prepared BERO at 50K. The wavelength used was 0.58889 Å.

To explore the fine structure characteristic of different samples, we conducted the synchrotron radiation XRD and further make the Rietveld refinements at 50K and 300K. To distinguish the degree of disorder of A site (the ideal Ru-based pyrochlores can be marked by $A_2Ru_2O_7$), we pay much attentions on the thermal parameters, which can be used to reflect the atom vibrations around the equilibrium positions. Apparently, the U_{iso}(Bi) in BRO is higher than U_{iso}(Er) in ERO whether at 50 K or 300 K, demonstrating that a higher degree of disorder happened in BRO than ERO and the 6s lone pair electrons of Bi^{3+} cations are responsible for the A-site disorder. Interestingly, the thermal parameters are differently changed in BRO and ERO as increasing temperature from 50 K to 300 K, because the thermal response to atomic disordered-hybridization of Er element is weaker than Bi that makes the changes in U_{iso}(Bi) become more drastic than U_{iso}(Er). Therefore, the symmetry breaking induced by atomic disordered-hybridization in pristine BRO sample can be effectively restricted by implanting some Er atoms, and the transition of U_{iso}(A-site) in BERO also can be easily understood from

this viewpoint. We can imagine that atomic occupation of Ru and O atoms will be changed correspondingly. Notably, the provided thermal parameters are isotropic, because a, b, c orientations are equivalent in the cubic structure that cannot give a reasonable reliability factors (R_p, R_{wp} (%) >10%)[4,5]. In addition, a little negative occupation at Bi site in pristine BRO at 300 K can be found, which only can be attributed to the disorder of Bi atoms[4]. In the whole, the atomic disordered-hybridization in BERO can be effectively alleviated by implanting Er atoms comparing to the BRO.

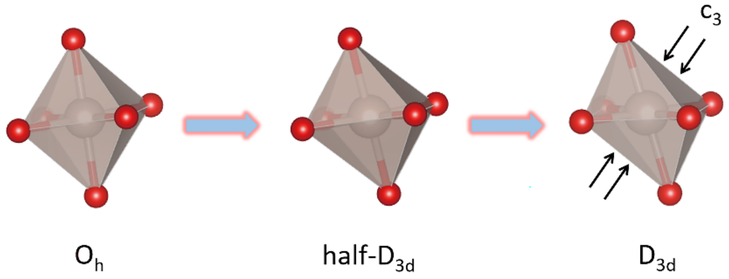

Supplementary Figure 5. Schematic illustrations of distorted RuO_6 structures.

Interestingly, the RuO_6 coordination octahedron will be changed as the degree of symmetry breaking and finally demonstrate a distinguishing physical characteristic. Generally, the ideal RuO_6 octahedron resulting a local point symmetry of Ru coordination is O_h, which is usually present in perovskite-based structures. Interestingly, the RuO_6 coordination polyhedron in BRO cannot be simply described as an ideal octahedron, because the interstitial Bi^{3+} cation with 6s long pair electrons will increase the Ru-O-Ru bond angle and finally lead to a distortion of octahedral RuO_6 coordination by trigonal compression along a three-fold axis. This local structural deformation will cause a symmetry transition from O_h to D_{3d}, as shown in Supplementary Figure 5 and Figure 1c. When some Bi atoms are replaced by Er atoms (sample BERO) to regulate the symmetry breaking and orbital splitting, the distorted octahedral RuO_6 coordination will be alleviated due to a smaller Ru-O-Ru bond angle in BERO compared with BRO, in which this controllable symmetry breaking in BERO demonstrate a half-disordered atomic configuration with D'_{3d} symmetry.

Furthermore, this symmetry-breaking-dependent structure is expected to reconfigure the orbital degeneracy and spin-related electron occupation. In a distorted RuO_6 octahedron (D_{3d} or D'_{3d} - symmetry), the partially filled t_{2g} orbitals will be further

degenerated to a 2-fold e_g band and a singlet a_{1g} band, which are described in Figure 1d. In addition, the catalytic mechanism about this symmetry breaking contribution is also provided in the former. In conclusion, the symmetry breaking transition from O_h and D_{3d} could be reflected by the changes in distorted RuO_6 octahedron and electronic band structure, which plays a determining role in efficient acidic OER.

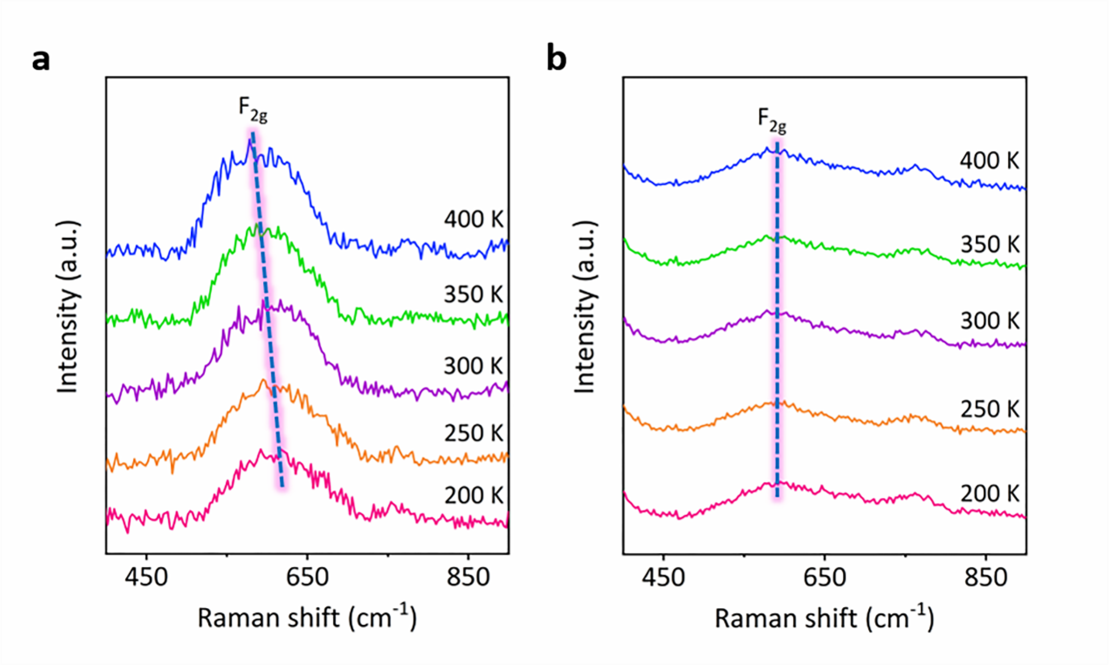

Supplementary Figure 6. Raman spectra of the (a) BRO and (b) ERO samples under different temperature.

The F_{2g} mode in BRO sample can be shifted as temperature but this change cannot occur at ERO sample.

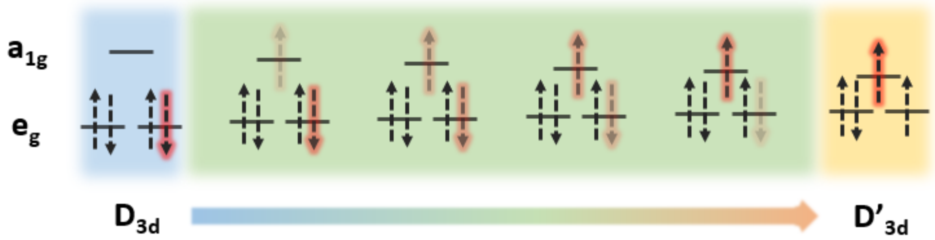

Supplementary Figure 7. The d-orbital splitting as symmetry transition.

When the RuO_6 coordination polyhedron in BERO is changed into D'_{3d} symmetry from D_{3d} point group, the splitting energy between e_g and a_{1g} orbital is slowly decreased. Therefore, the electrons at e_g orbitals can easily hop onto a_{1g} orbital due to electron-phonon interaction.

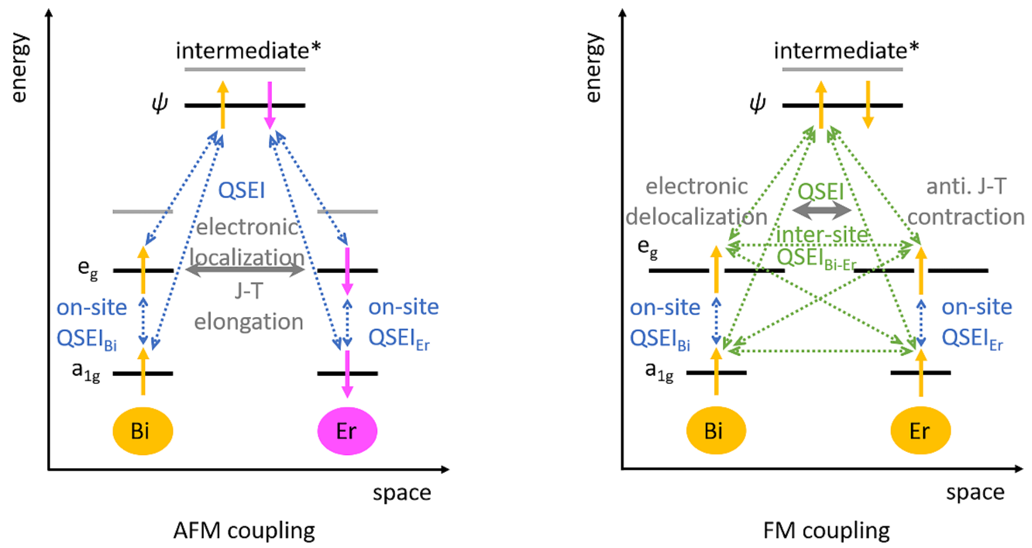

Supplementary Figure 8. The simplified QSEI energy plots for catalysts with predominant with AFM (left) and FM (right) couplings.

To further disclose the correlation between magnetic structure and OER activity, the spin energic terms depending on QSEI comprise two possible spatial interactions, as displayed in Supplementary Figure 8, where the extra on-site interactions takes place with the same atomic boundary and the extra interatomic interactions take place among different atoms. In the system with dominant AFM coupling, the strong intra-atomic QSEI will localize in space with 4d-5f pen shells that allow a stable low-spin orbital configurations by decreasing exclusively the on-site electronic configuration. It is important to note that no extra interatomic QSEI between AFM-coupled spin-configurations can be triggered and the interatomic Coulomb repulsions are intact in the catalyst. In this case, AFM Jahan-Teller elongations emerge to help reduce the electronic repulsions, which might appear along the direction of the highest occupied antibonding orbitals. Generally speaking, the OER reactive activity in AFM configuration will be reduced by the stabilization and additional localization of the

electron pairs, Fermi heaps between metal centers and the relative destabilization at the lowest unoccupied orbitals. This fact is likewise related with the enhanced electronic localization in space as shown in Figure 1b with lower symmetry, in which the number of electronic states at Fermi level for AFM configuration is reduced. This is because that the magnetic interactions can reduce the radial extension of the d orbitals and their electronic repulsions. The catalytic activity decreases especially for AFM Mott insulators, with the opening of a band gap between the occupied Mott lower valence band and empty upper conduction band. This can be used to explain why the OER performance in BRO and ERO samples are not better than BERO in Figure 4.

On the contrary, the total number of electrons with spin-up state is different than the number of spin-down electrons, as shown the right panel in Figure 1b, leading to the amount of interatomic QSEI center on the valence band creating Fermi holes that increasing the exchange spin-delocalization and decreasing the electronic repulsions. Strongly correlated itinerant electrons in FM coupling for the right panel in Supplementary Figure 8 have a profound contribution in catalysis, and they add spin-polarization to the thermodynamics. Based on the QSEI space-time mechanism, electrons with the same spin can exchange their orbitals more frequently. In this regard, their electronic repulsions reduce inside the catalysts, and this additional stabilizing energy together with empty valence orbitals yields to the enhanced catalytic activity in BERO sample via quantum interactions.

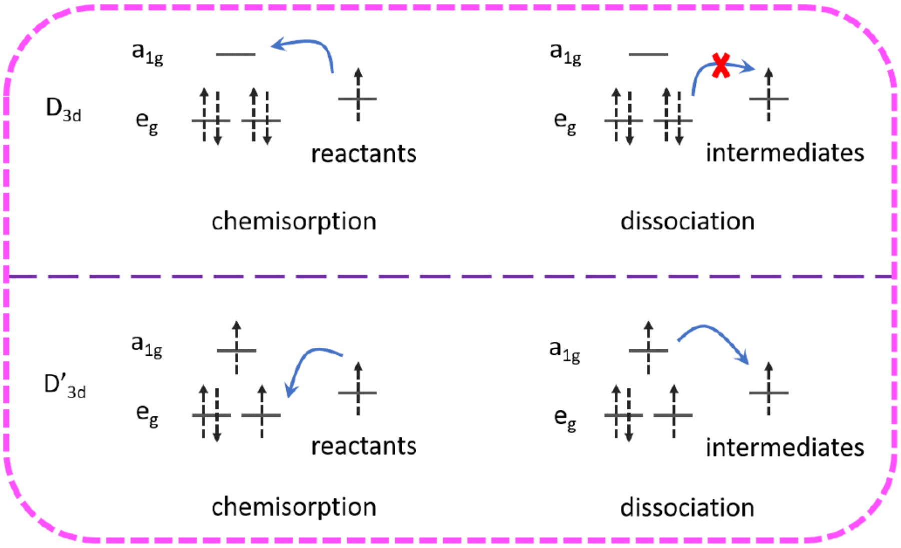

Supplementary Figure 9. Reaction mechanism design. The reaction mechanism for active site with different electronic occupation at different symmetry.

The efficiency of "acceptance-donation" process of electrons between the metal site and reactants is responsible for improving the OER catalytic activity, regulating the electronic occupation at d orbital is a feasible strategy to accelerate this process. Compared with the disordered atomic configuration (D_{3d}), the degenerated t_{2g} orbitals in D'_{3d} point group split into a half-filled a_{1g} band and e_g band, which are advantages to accelerate "acceptance-donation" process and improve the OER performance.

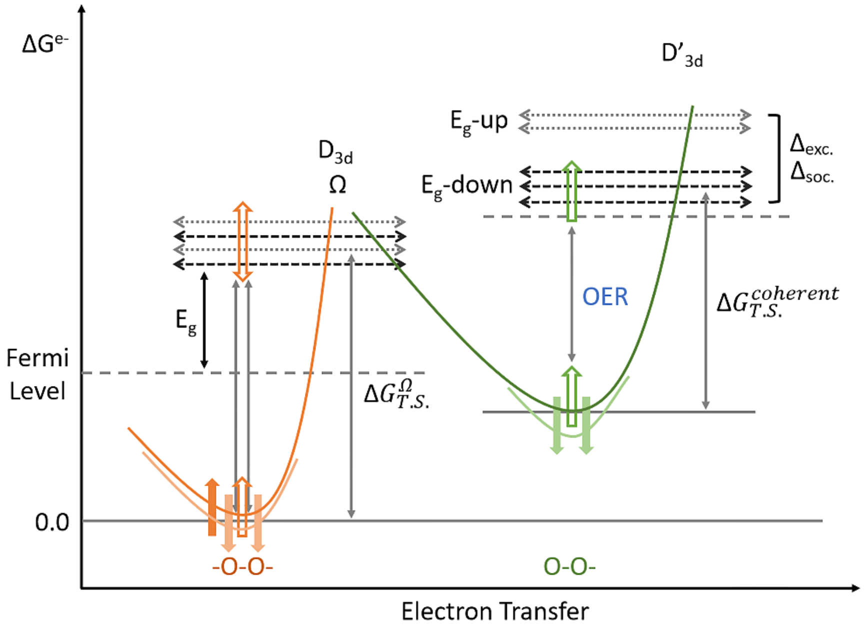

Supplementary Figure 10. Energy diagram representative of the OER via coherent spin transport.

To understand the reaction kinetics, we need to focus on the electronic configuration at the transition state (TS) during the electron transport, in which the nuclear wavepacket keeps unchanged due to the fast timescale for the electron transitions, allowing to the Franck-Condon principle. To demonstrate the schematic mechanism, the energy diagram representative of the last step in OER is displayed in Supplementary Figure 10. The band structure difference in D_{3d} symmetry at the ground state for the spin-up against the spin-down state is indispensable for fast spin tunneling, which cannot possess a spin polarization because of no unpaired electron at a_{1g} band. On the contrary, when the orbital splitting is changed in half-disordered structure with D'_{3d} symmetry, the e_g electrons can easily hop onto unfilled a_{1g} orbitals by quantum spin exchange interactions, leading to a FM configuration (see the magnetic characterization in Figure 3a-3c). In this case, this particular FM interaction can

accelerate the spin selective steps via weakening the Coulomb repulsions between electrons at the expense of the Coulomb attractions by the nuclei. Based on the schematic mechanism in Supplementary Figure 10, the rate coefficient of e-spin transfer towards the catalyst during OER can be described as equation (1), in which ΔH_{TS} and ΔS_{TS} is electronic enthalpy and entropy at transition state, respectively.

$$\mathrm{K(t)} \propto k_{st} \cdot e^{\frac{\Delta S}{k_B}} \cdot e^{\frac{-\Delta H}{k_B \cdot T}} \quad (1)$$

The OER cannot be triggered by vibrational oscillations but change in free energy at TS:

$$\Delta G = \Delta H - T\Delta S \quad (2)$$

As shown in Figure 5, the differentiated free energy for OER demonstrates that the catalysts with a high-spin state possess a higher catalytic activity. The e-spin transmission coefficient to reach the TS is k_{st}, which is strongly related with the electronic occupation at d orbitals. Compared to the D_{3d} point group, the non-bonding d orbitals in D'_{3d} symmetry makes an additional energy level generate at conduction band to act as a bridge, which agrees with the importance of covalent bonds in good conductors. The orbital splitting and electronic reoccupation for a_{1g} orbitals at Fermi level in $Bi_xEr_{2-x}Ru_2O_7$ sample can effectively increase the transmission coefficient, which can be confirmed by the difference in electrical conductivity in Figure 3f and EIS curves in Figure 4c.

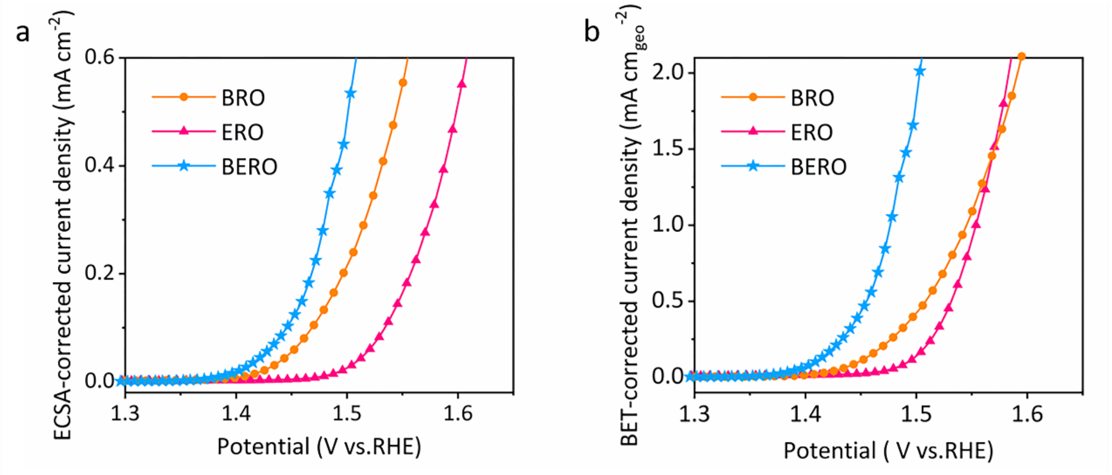

Supplementary Figure 11. The OER performance. (a) ECSA-normalized LSV curves of BRO, ERO and BERO catalysts. (b) BET-normalized LSV curves of BRO, ERO and BERO catalysts. The ECSA and BET values of different catalysts used here are listed in the Supplementary Table 5.

The compared results confirm that the OER performance for BERO sample is obviously better than BRO and ERO, which are independent of normalization methods. However, the slight changes in current values as normalization method cannot affect our conclusion. From this result, it is convincible to confirm that BERO catalyst with half-disordered hybridization has a higher intrinsic activity than BRO and ERO, in which the spin-related symmetry breaking plays a critical role in accelerating the catalytic kinetics.

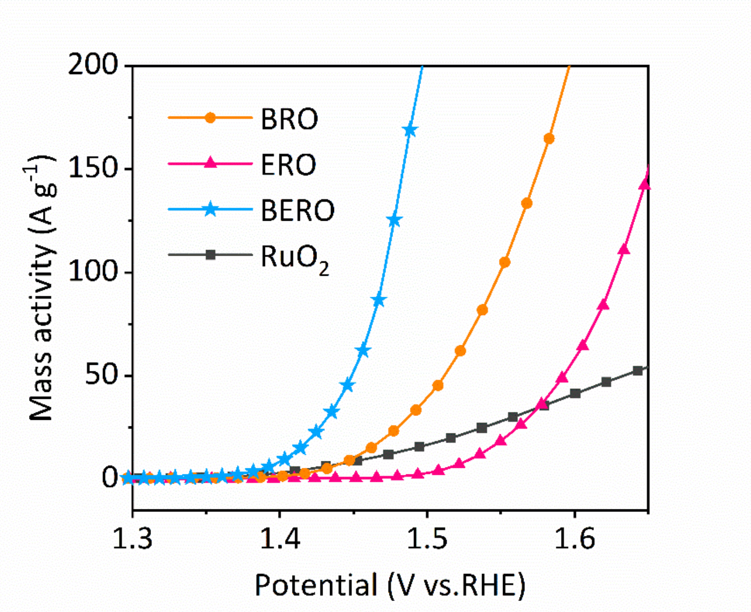

Supplementary Figure 12. Mass activity curves of the different samples under linear sweep voltammetry tests.

The amount of catalysts deposited on carbon paper (marked by CP) can be calculated by the quantity difference between catalysts@CP and CP:

$$\emptyset = \frac{\mathrm{m}(catalysts@CP)-\mathrm{m(CP)}}{\mathrm{S}} \tag{3}$$

Then the loading amount of catalysts on carbon paper for electrochemical tests are about 0.83±0.12 mg/cm^2. To exclude the contribution of loading amount difference onto catalytic performance, the mass activity curves are displayed in Supplementary Figure 12, in which the BERO sample also demonstrates the best reaction activity. We can conclude that the spin-related symmetry breaking induced by half-disordered hybridization in BERO plays the key role to accelerate the electrochemical reactive activity, which is independent of assessment method.

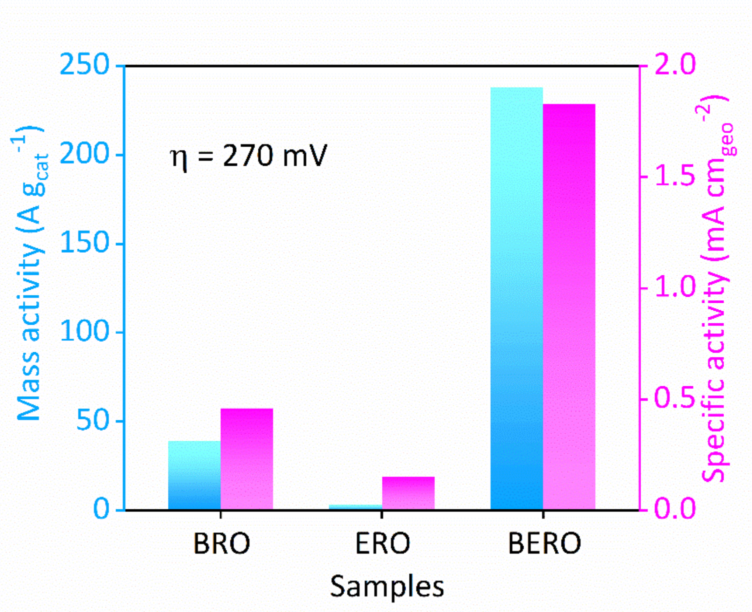

Supplementary Figure 13. Mass activity and BET surface area-normalized specific activity of BRO, ERO and BERO catalysts at η = 0.27 V (1.5V vs. RHE).

To display a fair comparison in OER performance, mass activity (normalized to the loading amount) and specific activity (normalized to the sample surface area as estimated from BET values) are obtained and shown in Supplementary Figure 13, respectively. Interestingly, BERO shows the excellent OER performance in acid, and the mass activity of BERO at 270 mV overpotential is about 6.1 and 74.4 times higher than that of BRO and ERO. Furthermore, the specific activity is also significantly higher than the other reports. The excellent OER activity only can be attributed to the spin-related symmetry breaking induced by half-disordered hybridization in BERO pyrochlores. In addition, the mass activity (normalized by catalysts) of BERO is superior to many reported state-of-the-art catalysts, as shown in Supplementary Table 14.

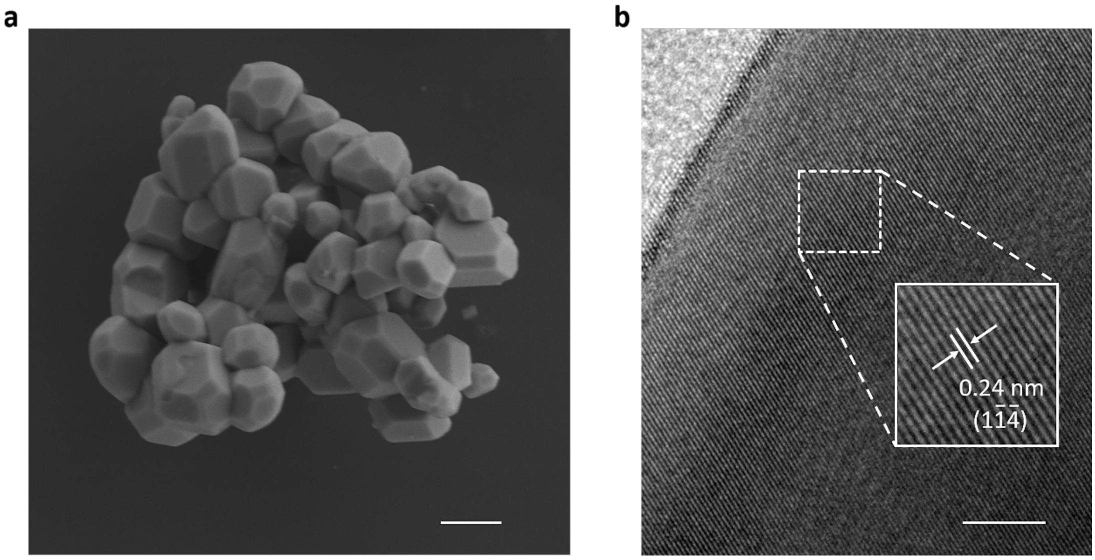

Supplementary Figure 14. The structural characterization. (a) The SEM image and (b) TEM images of BERO catalyst after OER test.

The stability of catalysts can be reflected by the microstructure of the catalysts that after OER test. The BERO catalyst maintains the original morphology and high crystallinity. No obvious amorphization can be observed by the TEM images.

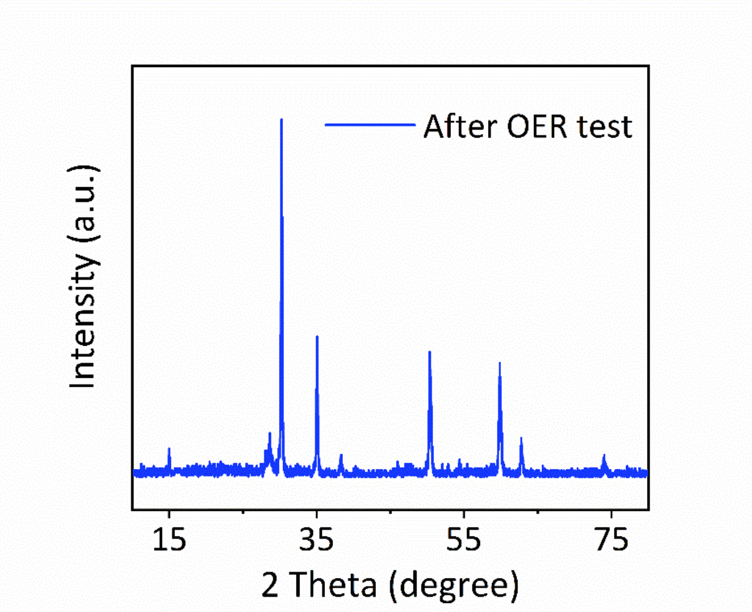

Supplementary Figure 15. XRD patterns of BERO catalyst after OER test.

Nor variation in XRD peaks can be observed after OER test, indicating there were no structural destruction.

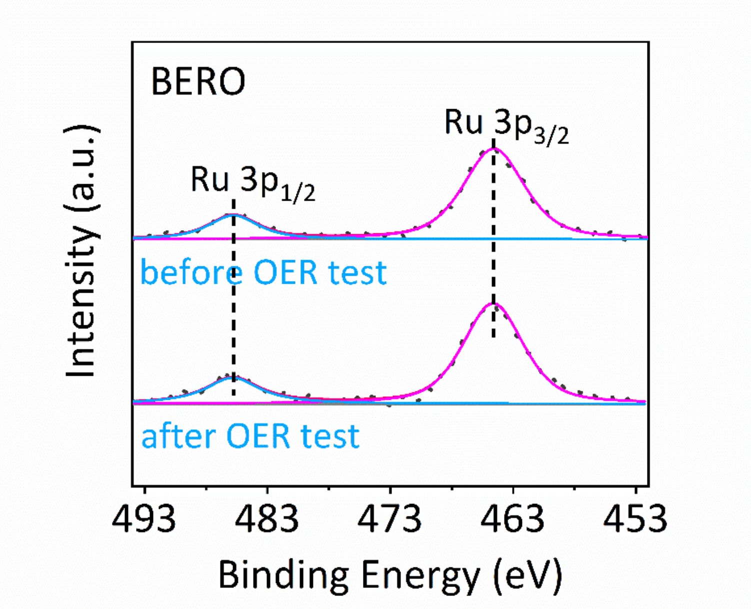

Supplementary Figure 16. XPS characterizations of BERO before and after OER test.

The high stability of BERO is also supported by XPS measurements after OER test. No visible changes in the positions of XPS core level peaks can be found on this sample before and after OER test.

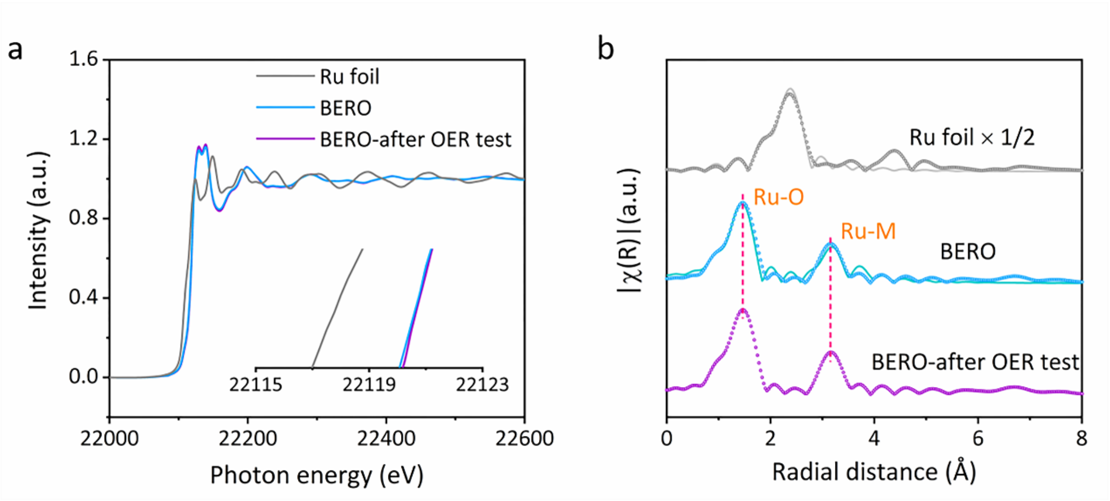

Supplementary Figure 17. X-ray absorption spectroscopy (XAS) results. (a) Ru K-edge XAS spectra of BERO before and after OER test. (b) Corresponding FT-EXAFS spectra.

To exclude the existence of Bi and Er cations leaching or small rutile-type particles, the X-ray absorption spectroscopy (XAS) measurements were conducted to reveal the stability of BERO after 100 hours OER test. As shown in Supplementary Figure 15a, the Ru K-edge XANES spectra of BERO before and after OER test demonstrate similar spectral features, and no distinctive energy shift can be observed as shown in the corresponding inset. To better understand the detailed electronic structure, the FT-EXAFS spectra are also provided in Supplementary Figure 15b to disclose the coordination environment of active sites after OER test. It is interesting to note that no obvious Ru-O and Ru-metal shells can be changed, indicating that no cation leaching or surface destruction occurs. In addition, the details fitting data are displayed in Supplementary Table 6. From the XAS comparison, we can conclude that our suggested BERO sample possesses an excellent OER stability in acid electrolytes.

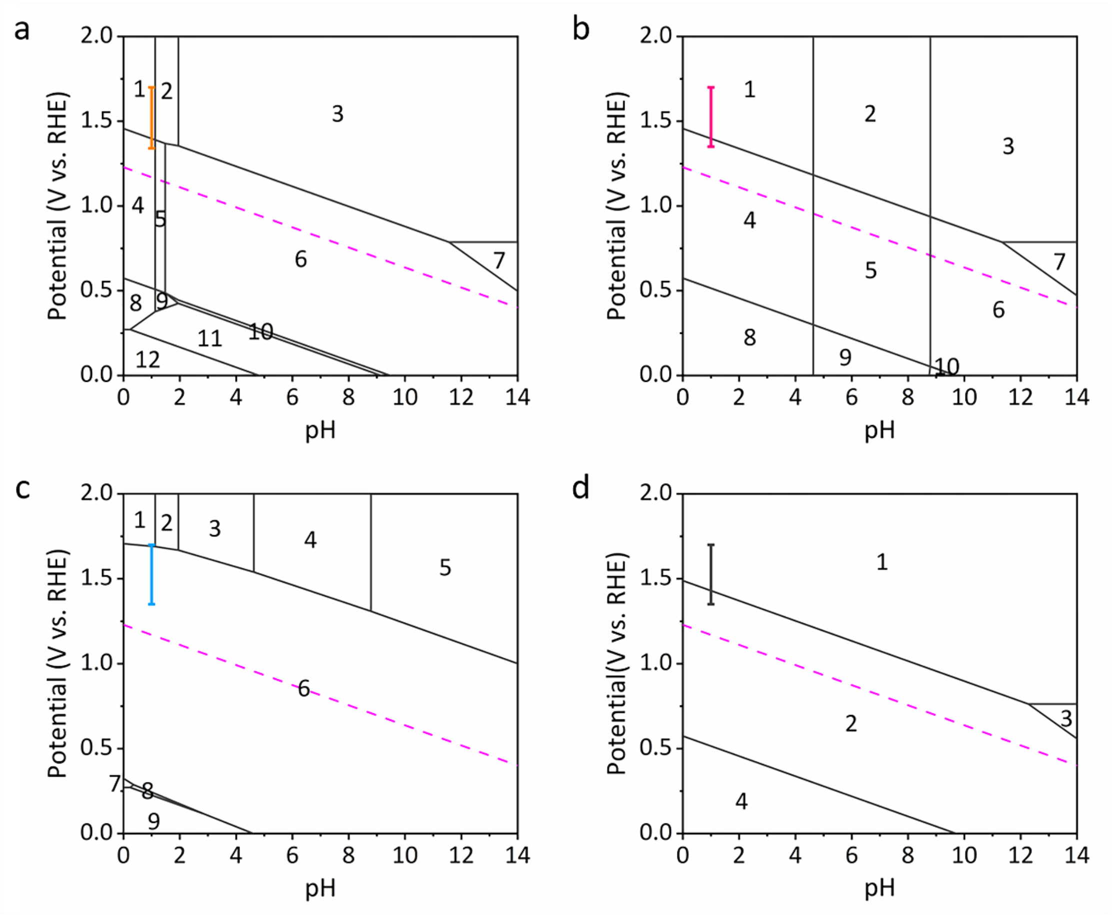

Supplementary Figure 18. Simulated structural stability. Calculated Pourbaix diagrams of (a) BRO, (b) ERO, (c) BERO and (d) RuO_2. The dashed pink lines in the Pourbaix diagram indicate the water oxidation potentials, whereas the colorful solid bar indicates the operating window tested in this work.

Pourbaix analysis are also conducted to reveal the structural stability of these catalysts. To confirm this point, the preferential phase under different electrochemical potentials and pH environment are obtained from the Pourbaix diagrams, as shown in Supplementary Table 9-12**.** The formation energies for different species, which may exist in these systems, were obtained by DFT predications combining with the Materials Project and Inorganic Crystal Structure Databases (ICSD). Pourbaix diagrams disclose that BRO, ERO and commercial RuO_2 demonstrate poor electrochemical stabilities at

oxygen evolution potentials (about 1.35 V ~ 1.70 V vs RHE) in 0.1 M $HClO_4$ conditions (PH=1). Generally speaking, the soluble RuO_4, RuO_2 and Bi^{3+} species will be the major phases under the OER windows in the BRO system as shown in Supplementary Figure 16a. Because, the transformation from Ru^{4+} to Ru>4^{+} at high voltages will lead to the decomposition of the pyrochlore, following by the dissolution of Bi element. This similar phenomenon also occurs at ERO and RuO_2 system, as shown in Supplementary Figure 16b. Remarkably, it is amazing to note that the stability region of BERO (Labels 6) is much larger than BRO, ERO and RuO_2, which can overlap the whole region of oxygen evolution potentials. This conclusion is consisting with our electrochemical tests, demonstrating the BERO with an excellent electrochemical stability.

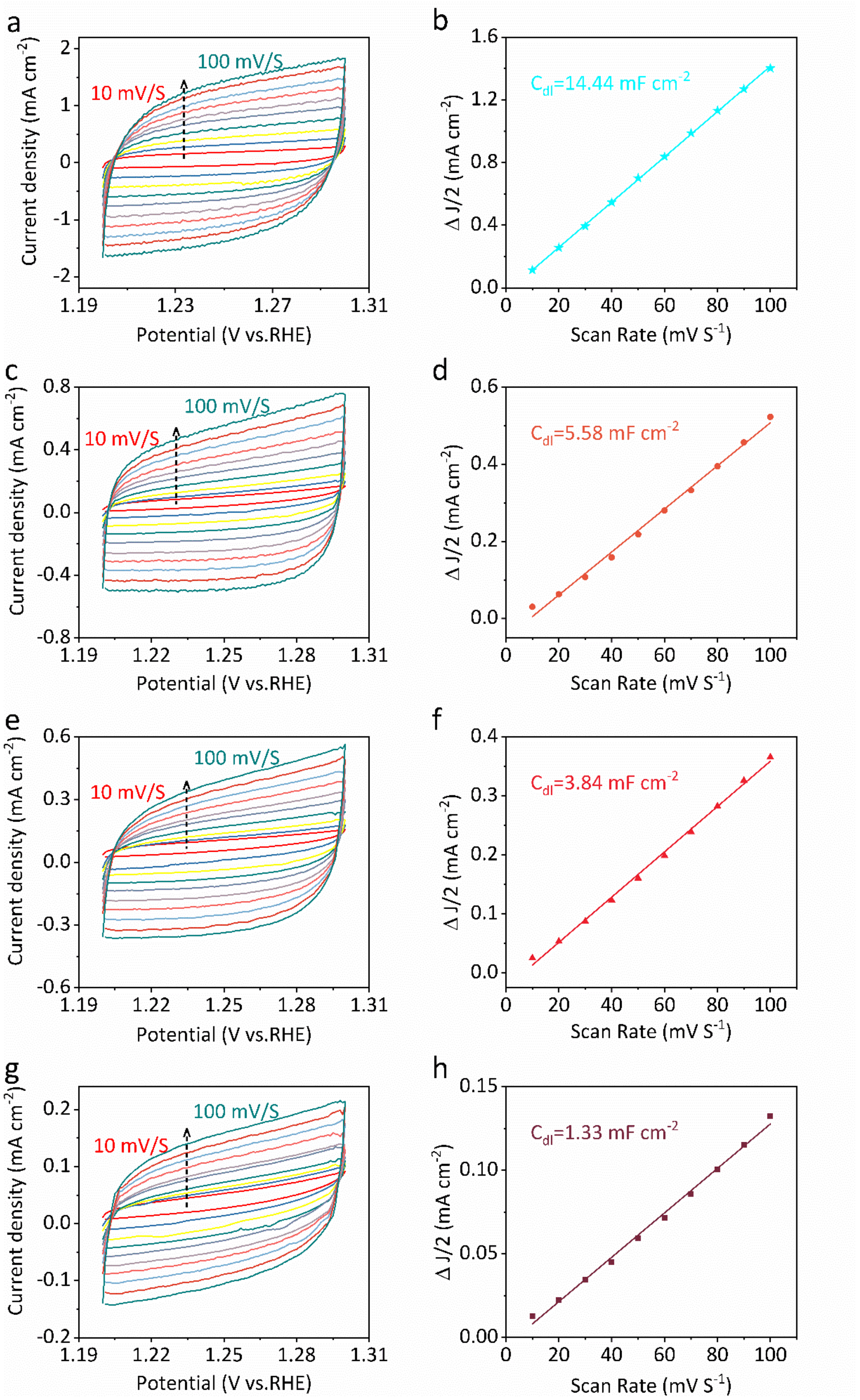

Supplementary Figure 19. The calculations about ECSA values. Cycle voltammetry (CV) measurements of (a) BERO, (c) BRO, (e) ERO and (g) RuO_2 catalysts from 1.20 V to 1.30 V (vs.RHE) at different scan rates. Linear fitting of the capacitive currents against CV scan rates for (b) BERO, (d) BRO, (f) ERO and

(h) RuO_2 catalysts.

They have a linear relationship between the electrochemical active surface area (ECSA) and double layer capacitance (C_{dl}) of the catalyst. In order to assess the ECSA of the different catalysts, C_{dl} of the catalysts were measured by a simple cyclic voltammetry method. Firstly, conducting the CV measurements in a none-faradic current region at scan rates of 10, 20, 30, 40, 50, 60, 70, 80, 90 and 100 mV S^{-1}. Secondly, a half of the measured capacitive current difference ($\Delta J/2$) at 1.25 V vs. Ag/AgCl was plotted against scan rate and double layer capacitance were determined from the slope of the linear fitting. The C_{dl} values for BERO, BRO and ERO are calculated to be 14.44, 5.58, 3.84 and 1.33 mF·cm^{-2}, respectively. Thirdly, the double layer capacitance is converted into an electrochemical surface area (ECSA) using the double layer capacitance value for a flat standard with 1 cm^2 of real surface area. We used the double layer capacitance (20-60 μF·cm^{-2}) of 40 μF·cm^{-2} here to calculate the ECSA

according to the equation (4):

$$\text{ECSA} = \frac{C_{dl}}{40\ \mu\text{F}/cm^2}\ \text{cm}^2{}_{\text{ECSA}} \qquad (4)$$

The ECSA values for BERO, BRO, ERO and RuO_2 are calculated to be 361.0 cm^2, 139.5 cm^2, 96.0 cm^2, and 33.3 cm^2, respectively.

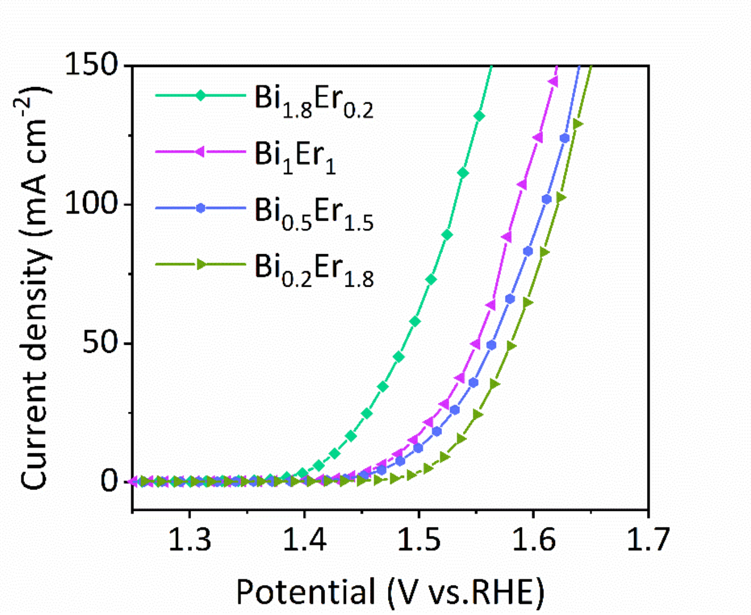

Supplementary Figure 20. The OER performance. Linear sweep voltammetry (LSV) curves of various $Bi_xEr_{2-x}Ru_2O_7$ with different disordered-hybridization. Including $Bi_{1.8}Er_{0.2}Ru_2O_7$, $Bi_{1.0}Er_{1.0}Ru_2O_7$, $Bi_{0.5}Er_{1.5}Ru_2O_7$ and $Bi_{0.2}Er_{1.8}Ru_2O_7$.

To explore the optimal OER performance in $Bi_xEr_{2-x}Ru_2O_7$ with different disordered-hybridization, the OER performance of various $Bi_xEr_{2-x}Ru_2O_7$ are compared and shown in Fig. 4a, Fig. 4f and Supplementary Fig. 20.

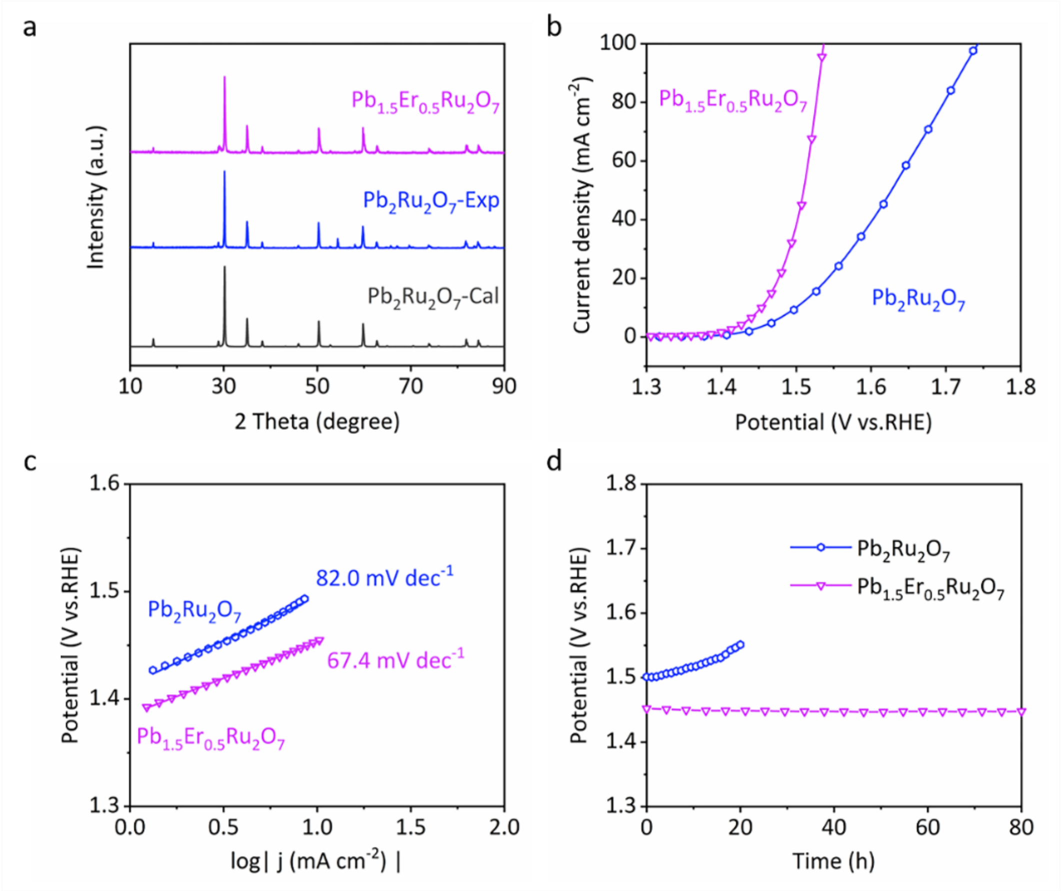

Supplementary Figure 21. The OER performance for PbEr-based catalysts. (a) X-ray diffraction (XRD) patterns of as-prepared $Pb_2Ru_2O_7$ and $Pb_{1.5}Er_{0.5}Ru_2O_7$ samples. (b) Polarization curves and (c) Tafel plots and (d) Stability test of the different samples.

Importantly, we further clarify this intriguing spin-related electronic reconfiguration induced by symmetry breaking is strongly related with the doping Er elements. Interestingly, both Bi and Pb element including lone-pair cations can lead to an atomic disorder, in which the degenerated d_{xy}, d_{xz} and d_{yz} orbitals in O_h symmetry split into a completely filled e_g band and an empty a_{1g} band for D_{3d} symmetry as shown in Figure 1b. However, the "acceptance-donation" cannot be realized easily to display a good catalytic activity, because the splitting energy between e_g and a_{1g} orbital is too

larger and valence electron cannot easily hop onto a_{1g} levels by electron-phonon interaction according to Goodenough-Kanamori rule. To realize this particular half-filled occupation at a_{1g} orbital, the Er cations, not sensitive to this symmetry breaking, should be introduced to control the atomic disorder degree in $Bi_xEr_{2-x}Ru_2O_7$ sample (named as half-disorder). As atomic disordering operation to eliminate orbital degeneracy, the spin electron occupation at a_{1g} and e_g orbitals will be reconfigured with symmetry-breaking-generated orbital splitting. In the view of this, the introduced Er plays a critical role in improving catalytic activity. As a universal law, the OER performances for $Pb_xEr_{2-x}Ru_2O_7$ are also conducted as shown in Supplementary Figure 19. The compared results also confirm that the doping Er element can enhance the OER performance in the $Pb_xEr_{2-x}Ru_2O_7$, similar to that of $Bi_xEr_{2-x}Ru_2O_7$. According to the similar method, the $Pb_2Ru_2O_7$ and $Pb_{1.5}Er_{0.5}Ru_2O_7$ samples are successfully fabricated, which can be confirmed by the XRD characterization as shown in Supplementary Figure 19a. The detailed LSV, Tafel and chronopotentiometry tests (Supplementary Figure 19b-d) demonstrate that the atomic-disordered $Pb_2Ru_2O_7$ also can be regulated by implanting some Er elements, leading to a higher catalytic activity. So, we can conduce that introduced Er cations, not sensitive to this symmetry breaking, play a critical role in controlling atomic disorder and OER performance. More interestingly, our suggested atomic half-disordering strategies in the multistate-hybridized Er-based catalysts could be foreseen in the follow-up researches to design more excellent acidic OER catalysts.

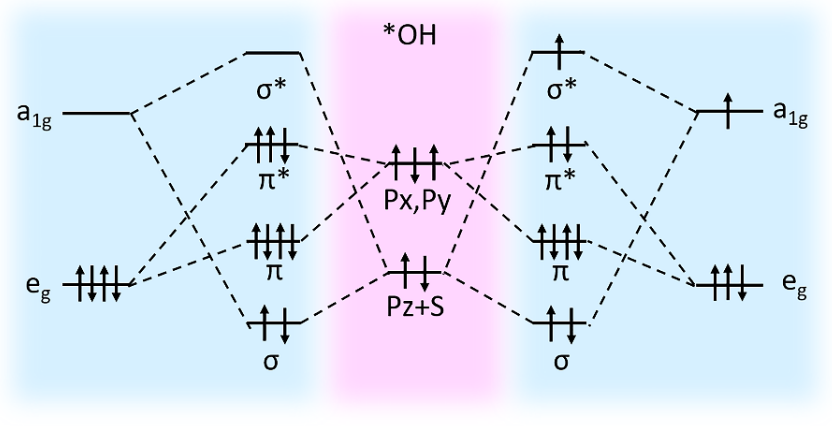

Supplementary Figure 22. The orbital interaction between active site and reactants.

To further understand the fundamental mechanism, the variation in enthalpy can be considered as two terms: equation 5, where Ea incorporates all the inherent molecular characteristics of the reaction, redox potential, adsorption energies and reorganization enthalpies, as displayed in Supplementary Figure 20.

$$\Delta H = Ea + Es \tag{5}$$

Es is the activation energy needed to create an itinerant TS in conduction band of the catalyst, which is relevant with conductivity[2]. For good catalysts, Ea and k_{st} should be comparable for similar reaction mechanisms and conditions. Theoretical studies on oxides surface conclude that the OER activity cannot be enhanced beyond noble diamagnetic metal oxides by tuning the binding energies (Ea). Hence, the decisive differences between good electro-catalysts appear due to changes in ΔS and Es, both sensitive to magnetic forces. Therefore, the presence of high-spin orbital configurations induced by reconfiguring orbital degeneracy in $Bi_xEr_{2-x}Ru_2O_7$ pyrochlores is responsible for the enhanced OER activity.

Supplementary Table 1. ICP result of BERO.

elements	Bi	Er	Ru
mg/L	139.30	38.42	91.04
mol ratio	1.48	0.51	2

Supplementary Table 2. Refinement data of $Bi_2Ru_2O_7$ (BRO) at 50 K and 300 K, with Bi at (1/2, 1/2, 1/2) site, Ru at (0, 0, 0) site, O_2 at (3/8, 3/8, 3/8) site. Constraints used for the refinement were n (Ru) = 2, and other negative occupations that less than 1% are marked by "~".

Sample BRO	50 K	300 K
a (Å)	10.26734	10.28377
n (Bi)	~ 2	~1.97
U_{iso} (Bi) $Å^2$	0.00451	0.00836
n (Ru)	2	2
U_{iso} (Ru) $Å^2$	0.00507	0.00616
x (O_1)	0.07985	0.07745
y (O_1)	-0.12500	-0.12500
z (O_1)	-0.12500	-0.12500
U_{iso} (O_1) $Å^2$	0.02730	0.02718
n (O_2)	~ 1	~ 1
U_{iso} (O_2) $Å^2$	0.00561	0.01098
R_p (%)	5.30	5.86
R_{wp} (%)	8.53	8.92

Supplementary Table 3. Refinement data of $Er_2Ru_2O_7$ (ERO) at 50 K and 300 K, with Er at (1/2, 1/2, 1/2) site, Ru at (0, 0, 0) site, O_2 at (3/8, 3/8, 3/8) site. Constraints used for the refinement were n (Ru) = 2, and other negative occupations that less than 1% are marked by "~".

Sample ERO	50 K	300 K
a (Å)	10.12534	10.13272
n (Er)	~ 2	~ 2
U_{iso} (Er) Å^2	0.00265	0.00447
n (Ru)	2	2
U_{iso} (Ru) Å^2	0.00286	0.00468
x (O_1)	0.34176	0.34265
y (O_1)	0.12500	0.12500
z (O_1)	0.12500	0.12500
U_{iso} (O_1) Å^2	0.01430	0.01820
n (O_2)	~ 1	~ 1
U_{iso} (O_2) Å^2	0.07412	0.11816
R_p (%)	6.11	5.51
R_{wp} (%)	8.53	7.89

Supplementary Table 4. Refinement data of $Bi_{1.5}Er_{0.5}Ru_2O_7$ (BERO) at 50 K and 300 K, with Bi/Er at (1/2, 1/2, 1/2) site, Ru at (0, 0, 0) site, O_2 at (3/8, 3/8, 3/8) site. Constraints used for the refinement were n (Ru) = 2, and other negative occupations that less than 1% are marked by "~".

Sample BERO	50 K	300 K
a (Å)	10.22156	10.23636
n (Bi)	~ 1.5	~ 1.5
n (Er)	~ 0.5	~ 0.5
U_{iso} (Bi/Er) Å^2	0.00285	0.00627
n (Ru)	2	2
U_{iso} (Ru) Å^2	0.00291	0.00621
x (O_1)	0.08234	0.08264
y (O_1)	-0.12500	-0.12500
z (O_1)	-0.12500	-0.12500
U_{iso} (O_1) Å^2	0.00967	0.01065
n (O_2)	~ 1	~ 1
U_{iso} (O_2) Å^2	0.01830	0.02530
R_p (%)	6.61	6.23
R_{wp} (%)	9.63	9.30

Supplementary Table 5. Comparison of ECSA and BET surface areas of the as-synthesized pyrochlore catalysts.

Catalysts	ECSA (cm^2, m^2/g)	BET surface area (m^2/g)
$B_2Ru_2O_7$ (BRO)	139.5 , 18.4	8.5
$Er_2Ru_2O_7$ (ERO)	96.0 , 11.3	2.1
$Bi_{1.5}Er_{0.5}Ru_2O_7$ (BERO)	361.0 , 45.0	11.5

Supplementary Table 6. Structural parameters of primitive BERO extracted from the EXAFS fitting (S_0^2 = 0.88, S_0^2 is the amplitude reduction factor, determined by the fitting of Ru foil. ΔE is the inner potential correction, ΔE for Ru foil is about -6.45 eV, and about -5.31 eV for BERO).

Sample	Path	CN[a]	R (Å)[b]	σ^2 ($Å^2$)[c]
Ru foil	Ru - Ru_1	6	2.67	0.00102
	Ru - Ru_2	6	2.64	0.00171
BERO	Ru - O	5.7	1.97	0.00267
	Ru - A site	6	3.68	0.00187
	Ru - Ru	6	3.84	0.00557

[a] CN is the coordination number.

[b] R is the bond length between Ru central atom and surrounding coordination atom.

[c] σ^2 is Debye-Waller factor.

Supplementary Table 7. Catalyst chemical stability in 0.1 M $HClO_4$ for 24 hours without applied potential. (BDL: below detection limits of the ICP-MS instrument)

Catalysts	Mass percent dissolved from catalysts (wt%)	
	Bi/Er-site	Ru
$Bi_2Ru_2O_7$ (BRO)	0.29%	BDL
$Er_2Ru_2O_7$ (ERO)	0.32%	0.03%
$Bi_{1.5}Er_{0.5}Ru_2O_7$ (BERO)	BDL	BDL
RuO_2 (commercial)	-	0.02%

Apart from the above results, the inductively coupled plasma mass spectrometry method (ICP-MS) was also conducted to further explore the surface stability of BERO in acidic electrolyte. To test the structural stability in harsh acid environment, we immerge our catalysts into 0.1 M $HClO_4$ for 24 hours and then detect the mass percent dissolved from the samples via ICP-MS method.

In details, we take out 10 mg catalyst and drop into a glass vial, then adding 5mL 0.1 M $HClO_4$, mixed, and allowed to sit undisturbed for 24 hours under Ar atmosphere protect. Subsequently, the solution was filtered and diluted with 5 mL Millipore water for ICP-MS tests. Finally, the total mass of each dissolved species are normalized by the total mass of the elements, according to $A_2Ru_2O_7$ stoichiometry. The results are collected and compared in Supplementary Table 7, in which no obvious cation leaching can be detected.

Supplementary Table 8. Catalyst electrochemical stability in 0.1 M $HClO_4$ during chronopotentiometry tests by maintaining the current density of 10 mA cm^{-2}.

Catalysts	Mass percent dissolved from catalysts (wt%)					
	10 hours		20 hours		100 hours	
	A-site	Ru	A-site	Ru	A-site	Ru
$Bi_2Ru_2O_7$	4.1%	3.7%	4.9%	4.6%		
$Er_2Ru_2O_7$	4.2%	3.3%	4.4%	3.9%		
$Bi_{1.5}Er_{0.5}Ru_2O_7$	BDL	BDL	BDL	BDL	2.2%&2.1%	1.7%
RuO_2	-	5.2%				

After that, the electrochemical stability under 10, 20, and 100 h OER conditions are assessed by ICP-MS tests and the collected results are shown in Supplementary Table 8. It can be observed that the Bi or Er and Ru elements are leached from BRO and ERO samples after 20 h chronopotentiometry test, but this phenomenon cannot occur at BERO sample. More interestingly, there is a little amount of metal cation dissolution (Bi: 2.2%; Er: 2.1%; Ru: 1.7%) after the BERO sample suffering 100 h chronopotentiometry test. These experiments undoubtedly confirm that the BERO catalysts display an excellent chemical stable under harsh acid OER conditions.

Supplementary Table 9. Domain Labels for BRO Pourbaix Diagram (Supplementary Figure 16a).

Labels	Species
1	$RuO_4 + Bi^{3+}$
2	$RuO_4 + BiOH^{2+}$
3	$RuO_4 + Bi_2O_3$
4	$RuO_2 + Bi^{3+}$
5	$RuO_2 + BiOH^{2+}$
6	BRO ($Bi_2Ru_2O_7$)
7	$RuO_4^{2-} + Bi_2O_3$
8	$Ru + Bi^{3+}$
9	$Ru + BiOH^{2+}$
10	$Ru + Bi_2O_3$
11	Ru + BiO
12	Ru + Bi

Supplementary Table 10. Domain Labels for ERO Pourbaix Diagram (Supplementary Figure 16b).

Labels	Species
1	$RuO_4 + Er^{3+}$
2	$RuO_4 + ErO^{+}$
3	$RuO_4 + Er(OH)_3$
4	$RuO_2 + Er^{3+}$
5	$RuO_2 + ErO^{+}$
6	ERO ($Er_2Ru_2O_7$)
7	$RuO_4^{2-} + Er(OH)_3$
8	$Ru + Er^{3+}$
9	$Ru + ErO^{+}$
10	$Ru + Er(OH)_3$

Supplementary Table 11. Domain Labels for BERO Pourbaix Diagram (Supplementary Figure 16c).

Labels	Species
1	$RuO_4 + Bi^{3+} + Er^{3+}$
2	$RuO_4 + BiOH^{2+} + ErO^{+}$
3	$RuO_4 + Bi_2O_3 + Er^{3+}$
4	$RuO_4 + Bi_2O_3 + ErO^{+}$
5	$RuO_4 + Bi_2O_3 + Er(OH)_3$
6	BERO ($Bi1.5Er_{0.5}Ru_2O_7$)
7	$Ru + Bi^{3+} + Er^{3+}$
8	$Ru + BiO + Er^{3+}$
9	$Ru + Bi + Er^{3+}$

Supplementary Table 12. Domain Labels for RuO_2 Pourbaix Diagram (Supplementary Figure 16d).

Labels	Species
1	RuO_4
2	RuO_2
3	RuO_4^{2-}
4	Ru

Supplementary Table 13. The comparison of OER performance in acid media between BERO and previously reported pyrochlores and Ru/Ir-based electrocatalysts.

Catalyst	Electrolyte solution	Overpotential (mV vs. RHE @10 mA cm^{-1})	Tafel slope (mV dec^{-1})	Stability test (h)	Reference
$Bi_{1.5}Er_{0.5}Ru_2O_7$	0.1 M $HClO_4$	180	51.3	100	This work
$Y_{1.8}Cu_{0.2}Ru_2O_7$	1 M H_2SO_4	-	52	6	6
$Y_2Ru_2O_{7-\delta}$	0.1 M $HClO_4$	190	55	8	7
Porous-$Y_2[Ru_{1.6}Y_{0.4}]O_7$	0.1 M $HClO_4$	~240	37	-	8
$Y_{1.85}Ba_{0.15}Ru_2O_7$	0.5 M H_2SO_4	270	40.2	12	9
$Y_{1.85}Zn_{0.15}Ru_2O_7$	0.5 M H_2SO_4	291	36.9	8	10
$Nd_2Ru_2O_7$	0.1 M $HClO_4$	~323	~40	10	11
$Y_{1.7}Sr_{0.3}Ru_2O_7$	0.5 M H_2SO_4	264	44.8	28	12
$Ir_{0.06}Co_{2.94}O_4$	0.1 M $HClO_4$	~290	45	200	13
3R-IrO_2	0.1 M $HClO_4$	188	52	511	14
$Cr_{0.6}Ru_{0.4}O_2$	0.5 M H_2SO_4	178	58	10	15
$CaCu_3Ru_4O_{12}$	0.5 M H_2SO_4	171	40	24	16
Ru-N-C	0.5 M H_2SO_4	267	52.6	30	17
C-Ultrafine defective RuO_2	0.5 M H_2SO_4	179	36.9	20	18
AlNiCoIrMo	0.5 M H_2SO_4	~230	55.2	45	19
$SrZrO_3$-$SrIrO_3$	0.1 M $HClO_4$	240	43	10	20

To demonstrate the advance in our suggested catalysts, the OER performances of these reported catalysts are collected and compared in Supplementary Table 13. The comparisons confirm that our suggested BERO catalysts have an obvious advantage comparing to these reported catalysts.

Supplementary Table 14. The comparison of mass activity in acid media between BERO and previously reported Ru/Ir-based electrocatalysts.

Catalyst	Electrolyte solution	Overpotential for mass activity value (mV)	mass activity (A g^{-1})	Reference
$Bi_{1.5}Er_{0.5}Ru_2O_7$	0.1 M $HClO_4$	270	238.0	This work
$Y_2Ru_2O_{7-\delta}$	0.1 M $HClO_4$	270	19.6	7
$Cr_{0.6}Ru_{0.4}O_2$	0.5 M H_2SO_4	270	229	15
IrCoNi PHNCs	0.1 M $HClO_4$	270	39.2	21
amorphous Ir NSs	0.1 M $HClO_4$	300	221.8	22
crystalline Ir NSs	0.1 M $HClO_4$	300	88.7	22
IrO_2-RuO_2@Ru	0.5 M H_2SO_4	270	13.2	23
Co-IrCu ONC	0.1 M $HClO_4$	270	170	24
IrOx-Ir	0.5 M H_2SO_4	270	28.2	25
$BaYIrO_6$	0.1 M $HClO_4$	270	10	26
IrNiCu DNF	0.1 M $HClO_4$	270	124.8	27

Supplementary Reference:

1. Gracia, J., Sharpe, R. & Munarriz, J. Principles determining the activity of magnetic oxides for electron transfer reactions. *J. Catal.* **361**, 331-338 (2018).
2. Gracia, J. Spin dependent interactions catalyse the oxygen electrochemistry. *Phys. Chem. Chem. Phys.* **19**, 20451 (2017).
3. Biz, C., Fianchini, M. & Gracia, J. Strongly correlated electrons in catalysis: focus on quantum exchange. *ACS Catal.* **11**, 22, 14249-14261 (2021).
4. Avdeev, M., Haas, M. K., Jorgensen, J. D., & Cava, R. J. Static disorder from lone-pair electrons in $Bi_{2-x}M_xRu_2O_{7-y}$ (M = Cu, Co; x = 0, 0:4) pyrochlores. *J. Solid State Chem.* **169**, 24-34 (2002).
5. Radosavljevic, I., Evans, J. & Sleight A. Synthesis and Structure of Pyrochlore-Type Bismuth Titanate. *J. Solid State Chem.* **136**, 63-66 (1998).
6. Kuznetsov, D. A. et al. Tailoring lattice oxygen binding in ruthenium pyrochlores to enhance oxygen evolution activity. *J. Am. Chem. Soc.* **142**, 7883-7888 (2020).
7. Kim, J. et al. High-performance pyrochlore-type yttrium ruthenate electrocatalyst for oxygen evolution reaction in acidic media. *J. Am. Chem. Soc.* **139**, 12076-12083 (2017).
8. Kim, J. et al. A porous pyrochlore $Y_2[Ru_{1.6}Y_{0.4}]O_{7-\delta}$ electrocatalyst for enhanced performance towards the oxygen evolution reaction in acidic media. *Angew. Chem. Int. Ed.* **57**, 13877-13881 (2018).
9. Feng, Q. et al. Influence of surface oxygen vacancies and ruthenium valence state on the catalysis of pyrochlore oxides. *ACS Appl. Mater. Interfaces* **12**, 4520-4530

(2020).

10. Feng, Q. et al. Highly active and stable ruthenate pyrochlore for enhanced oxygen evolution reaction in acidic medium electrolysis. *Appl. Catal. B: Environ.* **244**, 494-501 (2019).

11. Hubert, M. A. et al. Acidic oxygen evolution reaction activity-stability relationships in Ru-based pyrochlores. *ACS Catal.* **10**, 12182-12196 (2020).

12. Zhang, N. et al. Metal substitution steering electron correlations in pyrochlore ruthenates for efficient acidic water oxidation. *ACS Nano* **15**, 8537-8548 (2021).

13. Shan, J. Q. et al. Short-range ordered iridium single atoms integrated into cobalt oxide spinel structure for highly efficient electrocatalytic water oxidation. *J. Am. Chem. Soc.* **143**, 5201-5211 (2021).

14. Fan, Z. L. et al. Extraordinary acidic oxygen evolution on new phase 3R-iridium oxide. *Joule* **5**, 3221-3234 (2021).

15. Lin, Y. C. et al. Chromium-ruthenium oxide solid solution electrocatalyst for highly efficient oxygen evolution reaction in acidic media. *Nat. Commun.* **10**, 162 (2019).

16. Miao, X. B. et al. Quadruple perovskite ruthenate as a highly efficient catalyst for acidic water oxidation. *Nat. Commun.* **10**, 3809 (2019).

17. Cao, L. L. et al. Dynamic oxygen adsorption on single-atomic Ruthenium catalyst with high performance for acidic oxygen evolution reaction. *Nat. Commun.* **10**, 4849 (2019).

18. Ge, R. X. et al. Ultrafine defective RuO_2 electrocatayst integrated on carbon cloth for robust water oxidation in acidic media. *Adv. Energy Mater.* **9**, 1901313 (2019).

19. Jin, Z. Y. et al. Nanoporous Al-Ni-Co-Ir-Mo high-entropy alloy for record-high water splitting activity in acidic environments. *Small* **15**, 1904180 (2019).

20. Liang, X. et al. Perovskite-type solid solution nano-electrocatalysts enable simultaneously enhanced activity and stability for oxygen evolution. *Adv. Mater.* **32**, 2001430 (2020).

21. Feng, J. R. et al. Iridium-based multimetallic porous hollow nanocrystals for efficient overall-water-splitting catalysis. *Adv. Mater.* **29**, 1703798 (2017).

22. Wu, G. et al. A general synthesis approach for amorphous noble metal nanosheets. *Nat. Commun.* **10**, 4855 (2019).

23. Li, G. Q., Li, S. T., Ge, J. J., Liu, C. P. & Xing, W. Discontinuously covered IrO_2-RuO_2@Ru electrocatalysts for the oxygen evolution reaction: how high activity and long-term durability can be simultaneously realized in the synergistic and hybrid nano-structure. *J. Mater. Chem. A* **5**, 17221-17229 (2017).

24. Kwon, T. et al. Cobalt assisted synthesis of IrCu hollow octahedral nanocages as highly active electrocatalysts toward oxygen evolution reaction. *Adv. Funct. Mater.* **27**, 1604688 (2017).

25. Lettenmeier, P. et al. Nanosized IrO_x-Ir catalyst with relevant activity for anodes of proton exchange membrane electrolysis produced by a cost-effective procedure. *Angew. Chem. Int. Ed.* **128**, 752-756 (2016).

26. Diza-Morales, O. et al. Iridium-based double perovskites for efficient water oxidation in acid media. *Nat. Commun.* **7**, 12363 (2016).

27. Park, J. et al. Iridium-based multimetallic nanoframe@nanoframe structure: an

efficient and robust electrocatalyst toward oxygen evolution reaction. *ACS Nano* **11**, 5500-5509 (2017).
